# Dendritic inhibition differentially regulates excitability of dentate gyrus parvalbumin-expressing interneurons and granule cells

**DOI:** 10.1038/s41467-019-13533-3

**Published:** 2019-12-05

**Authors:** Claudio Elgueta, Marlene Bartos

**Affiliations:** Institute for Physiology I, Cellular and Systemic Neurophysiology, Medical Faculty of the University of Freiburg, 79104 Freiburg, Germany

**Keywords:** Cellular neuroscience, Biophysical models

## Abstract

Fast-spiking parvalbumin-expressing interneurons (PVIs) and granule cells (GCs) of the dentate gyrus receive layer-specific dendritic inhibition. Its impact on PVI and GC excitability is, however, unknown. By applying whole-cell recordings, GABA uncaging and single-cell-modeling, we show that proximal dendritic inhibition in PVIs is less efficient in lowering perforant path-mediated subthreshold depolarization than distal inhibition but both are highly efficient in silencing PVIs. These inhibitory effects can be explained by proximal shunting and distal strong hyperpolarizing inhibition. In contrast, GC proximal but not distal inhibition is the primary regulator of their excitability and recruitment. In GCs inhibition is hyperpolarizing along the entire somato-dendritic axis with similar strength. Thus, dendritic inhibition differentially controls input-output transformations in PVIs and GCs. Dendritic inhibition in PVIs is suited to balance PVI discharges in dependence on global network activity thereby providing strong and tuned perisomatic inhibition that contributes to the sparse representation of information in GC assemblies.

## Abstract (web summary)

Fast-spiking parvalbumin-expressing interneurons (PVIs) and granule cells of the dentate gyrus receive layer-specific dendritic inhibition. The authors show that distal and proximal dendritic inhibition differentially control input-output transformations in PVIs and granule cells.

## Introduction

Cognitive processes emerge from input−output transformations in cortical networks, which depend on the microcircuit connectivity and the dendritic tree as the biophysical substrate for synaptic input integration^1–5^. Input−output transformations are controlled by dendritic inhibition provided by GABAergic inhibitory interneurons^6–10^. How dendritic inhibition influences integration of excitatory signals has been mainly studied in cortical and hippocampal pyramidal cells^11–19^; however, much less is known on this process in GABAergic interneurons and glutamatergic GCs of the dentate gyrus. This is an important question because these neurons display distinct morphological and physiological properties^20–24^ compared to neocortical and hippocampal pyramidal cells and their in vivo activity stays under tight inhibitory control^25,26^.

PVIs and GCs of the dentate gyrus receive information from the entorhinal cortex via the perforant path and from the contralateral hippocampus via the commissural path^27^. Neuronal activity in this region is sparse, with only few GCs being active when rodents explore the environment^28–33^, and is under the control of various GABAergic interneuron types^30^. PVIs provide perisomatic inhibition onto large GC populations^34,35^, while dendritic inhibition originates mainly from hilar commissural path-associated interneurons (HICAPs), with axon collaterals in the inner molecular layer, and somatostatin (SOM)-expressing hilar perforant path-associated interneurons (HIPPs), with axons localized in the outer half of the molecular layer^36–39^. The size of GC assemblies appears to be under control of dendrite-inhibiting SOM-positive interneurons^40^ (SOMIs), which form numerous synapses at distal GC dendrites^41^. PVIs are strongly recruited by layer-specific excitatory inputs and receive HIPP-mediated distal feedback and HICAP-mediated proximal feedforward and feedback dendritic inhibition^38^. Due to their steep current−frequency relationship, even small changes in excitatory input strength will influence PVI recruitment and in turn their impact on information processing in the dentate gyrus^42,43^. Thus, dendritic inhibition may serve as a regulator of excitability, synaptic plasticity and sparse activity in the dentate gyrus^40,44,45^.

Previous computational and experimental investigations explored the interference of dendritic inhibition with Ca^2+^ signals in hippocampal and cortical principal cell dendrites evoked by back propagating action potentials^12,17,18,46^ and dendritic spike generation in vitro^11,15^ or sensory stimulation in vivo^47^, and showed that its impact is spatially and temporally confined. Much less is known regarding the effects of dendritic inhibition on the input−output transformations in interneurons and dentate gyrus principal cells. In this study we therefore asked: how does location, amplitude and timing of dendritic inhibition control excitatory input strength and action potential generation in PVIs compared to GCs in the dentate gyrus? By combining experimental and computational approaches, we show that in PVIs, off-path distal inhibition is more efficient than on-path proximal inhibition in controlling the amplitude of subthreshold excitatory postsynaptic signals (EPSPs) and similarly capable of silencing PVIs. These observations can be explained by a nonuniform distribution of the reversal potential of GABA_A_ receptor (GABA_A_Rs)-mediated signals (*E*_GABA_), K-Cl cotransporters and the density of GABA_A_R-mediated conductances (*G*_GABA_) along the somato-dendritic axis, resulting in weak shunting on-path and strong hyperpolarizing off-path inhibition. In contrast, in GCs we observed hyperpolarizing inhibition along the entire somato-dendritic tree and no *G*_GABA_ gradient, resulting in on-path inhibition being more efficient in silencing GCs than off-path inhibition.

## Results

### Cell type-dependent on- and off-path inhibitory efficiency

**Fig. 1:**
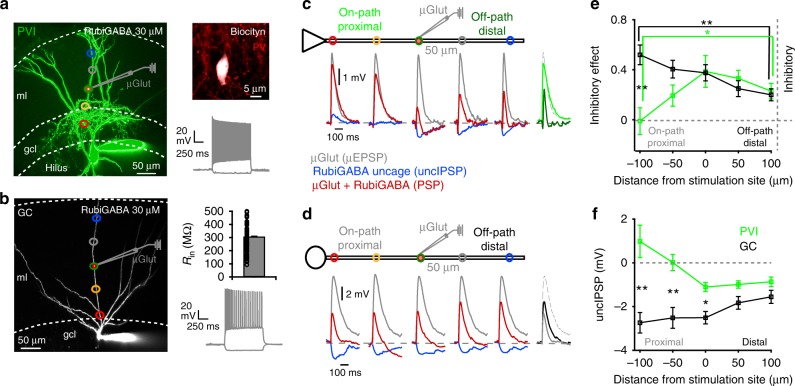
Different efficiency of on- and off-path inhibition in PVIs and GCs. **a**, **b** Left, two-photon image stack of a PVI (**a**) and a GC (**b**) loaded with Alexa Fluor-488. Somatic whole-cell recordings were performed while RubiGABA was uncaged at one out of five locations (colored circles) relative to the glutamate microiontophoresis (µGlut) stimulation site (scale bar 50 µm). Right upper, PV antibody-labeling of the cell shown on the left and its fast-spiking phenotype (−100 and 800 pA, 1 s, 233.6 Hz). Right lower, bar graph summarizing input resistances (*R*_in_) for 94 recorded GCs. Circles represent individual data points. Bottom, firing pattern of the GC shown on the left (−50 and 400 pA, 1 s, 30.9 Hz). **c**, **d** Schematic of a PVI and GC cell body (triangle and circle, respectively) with one apical dendrite. EPSPs were evoked by glutamate microiontophoresis (µEPSPs) at the middle apical dendrite of PVIs and GCs (154.5 ± 7.3 and 143.1 ± 8.7 µm distance from the soma, respectively; six PVIs, ten GCs, represented by µGlut pipette). GABA was uncaged at five locations (uncIPSP) relative to the µGlut position (50 µm steps). Superimposed traces show corresponding control µEPSPS (gray), IPSPs evoked by RubiGABA uncaging (uncIPSPs, blue) and PSPs resulting from the interaction of µEPSPs and uncIPSPs at the corresponding five uncaging locations (red). Superimposed traces on the right show µEPSP (gray dashed) and PSPs resulting from µEPSPs and uncIPSPs interaction at on-path (−100 µm; PVI bright-green, GC gray trace) and off-path (+100 µm; PVI dark green, GC black trace) uncaging locations. **e** Inhibitory effect calculated as 1 − (PSP amplitude/µEPSP amplitude) is plotted against uncaging location. Note on-path inhibition has a significantly larger effect on µEPSPs in GCs compared to PVIs (black vs. green lines, respectively), and off-path is more efficient than on-path inhibition in PVIs. **f** Mean uncIPSP amplitudes are plotted as a function of distance from µGlut stimulation site in PVIs (green) and GCs (black). Squares and bars with lines represent mean ± s.e.m. *p ≤ 0.05; **p ≤ 0.01. ml molecular layer, gcl granule cell layer.

The effect of dendritic inhibition on excitatory signals was examined using whole-cell patch clamp recordings from PVIs and GCs in acute slice preparations of the rat dentate gyrus (Fig. 1). The recorded interneurons had classical PVI properties. They discharged at high average frequency (226.9 ± 10.0 Hz), had low input resistances (*R*_in_ = 101.5 ± 6.1 MΩ, mean ± s.e.m.) and axon collaterals restricted to the granule cell layer (51 cells identified during two-photon imaging; Fig. 1a; 36 out of 64 cells identified post-hoc as basket or axo-axonic cells^36,37^; Supplementary Fig. 1). Antibody-labeling in a subset of recorded cells confirmed their PV-nature (17 out of 19 tested cells; Fig. 1a, Supplementary Fig. 1). In contrast, GCs discharged at lower average frequency (46.7 ± 4.5 Hz; *p* < 0.001; 84 cells, Fig. 1b, right) and had higher *R*_in_ (308.7 ± 11.4 MΩ; *p* < 0.001; 94 GCs; Fig. 1b, right). Thus, all recordings were obtained from fast-spiking interneurons, to which we refer as PVIs, and mature GCs.

To examine how the induction site of inhibitory signals influences EPSP size, we paired individually evoked EPSPs with IPSPs (Fig. 1c, d). Focal EPSPs were induced by glutamate microiontophoresis (µEPSPs) at one apical dendrite from PVIs or GCs (~150 µm to the recorded soma) and IPSPs were induced by uncaging RubiGABA (30 µM; uncIPSPs, Supplementary Fig. 2a, b) along the same apical dendrite either on-path, i.e. between the soma and the µEPSP induction site, “on-site”, at the location of µEPSP induction, or off-path, i.e. distal to the µEPSP induction point (Fig. 1a−d). UncIPSPs evoked by RubiGABA uncaging were blocked by SR-95531 revealing their GABA_A_R-mediated nature (Supplementary Fig. 2c). Cells were filled with Alexa Fluor-488 for precise positioning of uncaging spots and the µGlut pipette close to the dendrite using two-photon imaging (Fig. 1a, b). Excitatory signals in PVIs had smaller amplitudes and faster kinetic properties than in GCs (µEPSP amplitude 4.3 ± 0.7 vs. 7.3 ± 0.6 mV; half-width: 37.9 ± 7.2 vs. 92.7 ± 11.1 ms; six PVIs and ten GCs; *p* = 0.01 and 0.002 respectively, two-tailed Wilcoxon rank sum test; Fig. 1c, d, gray traces), equivalent to the simultaneous activation of ~4 and ~7 distally located glutamatergic synaptic inputs, respectively^21,23,48^. *E*_GABA_ was kept close to values previously described for dentate gyrus PVIs and GCs, by loading cells during recordings with a pipette solution that resulted in a chloride reversal potential of −63.3 mV^49,50^. The impact of inhibition on excitation was quantified as the inhibitory effect (IE **=** 1 − (PSP/µEPSP)). Inhibitory efficiency was the highest in PVIs when GABAergic signals were evoked on-site (0.39 ± 0.13, six cells; Fig. 1c, e), under conditions of a focal GABA_A_R-mediated conductance change (spatial extent < 20 µm). Unexpectedly, inhibition was still highly efficient when evoked 50 or 100 µm off-path from the µEPSP induction location (IE: 0.33 ± 0.06 and 0.23 ± 0.06; Fig. 1c, e), but markedly declined when GABAergic signals were evoked on-path, −50 or −100 µm proximal to the µEPSP induction site (−100 µm distance: IE = −0.01 ± 0.1, six cells; *p* = 0.04, two-tailed paired *t* test; Fig. 1e). Thus, the inhibitory effect increased from proximal to distal along the somato-dendritic axis of PVI apical dendrites to reach maximal values at distal branches (Fig. 1e). A contrasting picture emerged for GCs. Here, the inhibitory efficiency was highest for on-path (0.48 ± 0.08 at −100 µm distance from µEPSP induction site) and monotonically declined along the apical dendrite, being lowest for off-path inhibition (0.24 ± 0.05 at 100 µm distance; ten GCs; *p* = 0.005, two-tailed Wilcoxon signed-ranks test; Fig. 1d, e).

Taken together, PVIs and GCs show opposing nonuniform inhibitory efficiencies along the somato-dendritic axis. In PVIs excitatory signals evoked at the level of the medial perforant path were most efficiently reduced by distal off-path and on-site inhibition, whereas in GCs by proximal on-path GABAergic signals.

### Opposite somato-dendritic *E*_GABA_ gradients in PVIs and GCs

What factors may underlie the different dendritic inhibitory efficiency between PVIs and GCs? In PVIs the polarity of somatically recorded uncIPSPs depended on the RubiGABA uncaging location (Fig. 1c, blue traces). GABAergic signals were depolarizing if evoked close to the soma but hyperpolarizing at distal dendrites (1.0 ± 0.7 and −0.9 ± 0.2 mV for −100 and +100 µm distance to µEPSP induction site, respectively; six PVIs, *p* = 0.047, two-tailed Wilcoxon signed-ranks test; mean membrane potential −65.2 ± 0.6 mV; Fig. 1f). In contrast, GCs showed always hyperpolarizing responses independent of uncaging location, with significantly larger amplitudes for uncIPSPs evoked on- than off-path (−2.7 ± 0.5 and −1.6 ± 0.3 mV at −100 and +100 µm uncaging sites, respectively; 11 GCs; *p* = 0.21, two-tailed Wilcoxon signed-ranks test; mean membrane potential −69 ± 0.4 mV; Fig. 1f). Thus, *E*_GABA_ may be nonuniformly distributed along PVI dendrites.

**Fig. 2:**
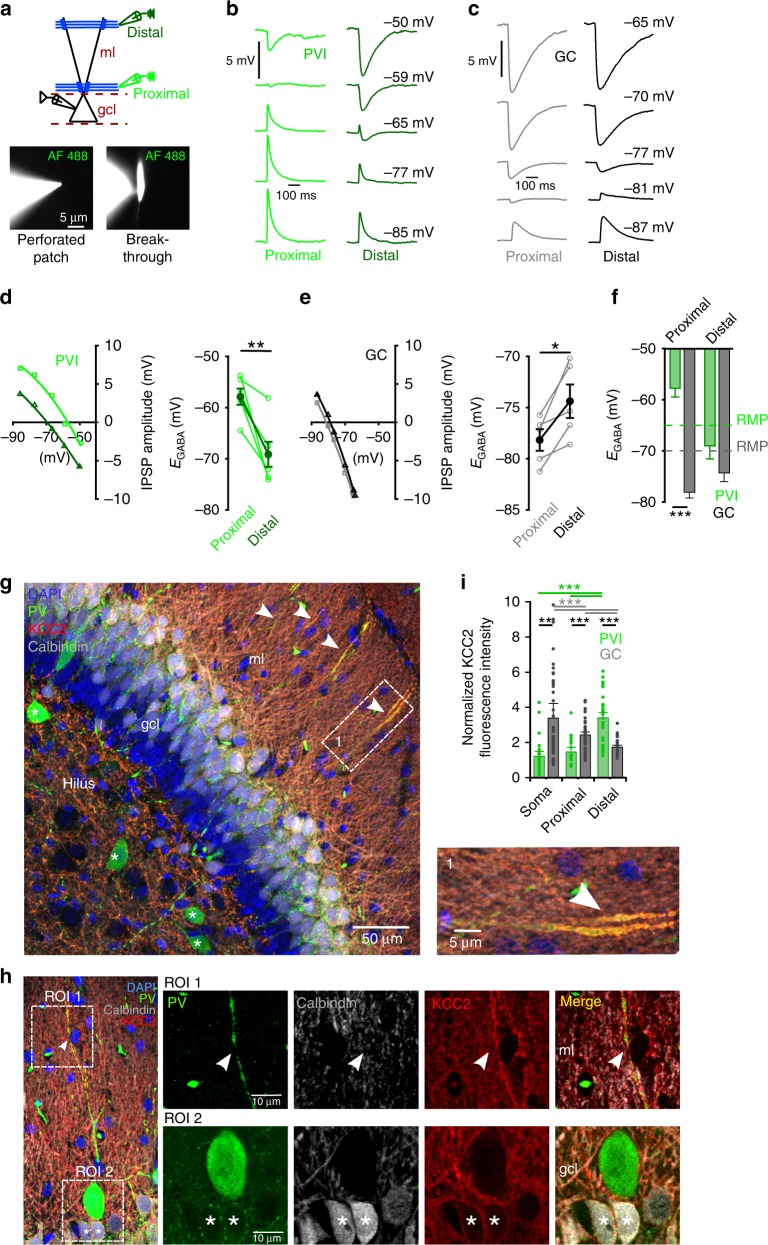
Opposite somato-dendritic *E*_GABA_ gradients in PVIs and GCs. **a** Schematic representation of the experimental design. Pharmacologically isolated GABA_A_R-dependent signals evoked by stimulation of the inner or the outer molecular layer (ml) were recorded using Gramicidin-A perforated-patch in PVIs or GCs. Disruption of the perforated-patch was evaluated by presence of Alexa Fluor-488 (AF 488) in the cell. **b**, **c** IPSPs evoked by proximal or distal stimulation were recorded at varying holding potentials in PVIs (**b**, bright and dark green traces, respectively) and GCs (**c**, gray and black traces, respectively). **d**, **e** Left, IPSP amplitude to voltage relationship for proximally and distally evoked IPSPs in a representative PVI (**d**) and GC (**e**). Right, graphs summarize *E*_GABA_ of proximally and distally evoked IPSPs in PVIs (**d**) and GCs (**e**). Open circles connected by lines represent data from individual cells, filled circles with lines represent means ± s.e.m. (five PVIs, five GCs). **f** Summary bar graph of *E*_GABA_ of proximally and distally evoked IPSPs in PVIs (green) and GCs (gray). Dashed lines represent corresponding resting membrane potentials. **g** Maximum intensity projection from 5 × 0.67 µm optical sections of a rat ventral dentate gyrus slice stained for parvalbumin (PV), KCC2 and calbindin, and counterstained with DAPI (scale bar 50 µm). Note that in the granule cell layer (gcl) membrane-bound KCC2 is expressed at the soma of calbindin-positive GCs but not in immature calbindin-negative GCs and rarely in PV-interneurons (asterisks), while PV-labeled dendrites in the molecular layer demonstrate strong expression of the cotransporter (arrowheads). Inset lower right, magnified section depicted by the white square (scale bar 5 µm). **h** Maximum intensity projection of 11 × 0.36 µm optical sections. ROI-1 and −2 are shown at higher magnification in the right panels (scale bars 10 µm). Note, KCC2 is expressed in calbindin-positive GC somata (asterisks) but not in the PVI (ROI 2), while distal dendrites of the same PVI show strong KCC2 staining (ROI 1, arrowhead). **i** Bar graphs summarize normalized fluorescence intensity of KCC2 antibody labeling at the soma, proximal and distal dendrites of PVIs and calbindin-expressing GCs. *p ≤ 0.05; **p ≤ 0.01; ***p ≤ 0.001.

To test this assumption we determined *E*_GABA_ (Fig. 2). To preserve the intracellular chloride concentration we performed Gramicidin-A somatic perforated-patch recordings. Pharmacologically isolated IPSPs were evoked by extracellular stimulation of the inner or outer molecular layer corresponding to proximal and distal GABAergic synaptic inputs (Fig. 2a−c). We recorded IPSPs at incremental membrane potentials and fit the IPSP amplitude to voltage relationships with a polynomial function (Fig. 2d, e). *E*_GABA_ values in PVIs were always more positive than the resting membrane potential at proximal sites, but more negative for distally evoked signals (*E*_GABA_ −57.9 ± 1.6 vs. −69.1 ± 2.4 mV, respectively; eight PVIs; *p* = 0.0016, two-tailed paired *t* test; Fig. 2b, d, f). In the following, we will refer to these forms of inhibition as “shunting” and “hyperpolarizing”, respectively. In contrast, GCs displayed *E*_GABA_ values more negative than the resting membrane potential, with *E*_GABA_ being significantly more negative for IPSPs evoked at proximal than at distal dendrites (−78.2 ± 1.0 vs. −74.4 ± 1.6 mV; six GCs; *p* = 0.02, two-tailed paired *t* test; Fig. 2c, e, f). Comparing the mean *E*_GABA_ values between the two cell types revealed significant differences for proximally but not for distally evoked GABA_A_R-mediated signals (*p* = 0.0001 and 0.17, respectively; two-tailed unpaired *t* test; Fig. 2f).

The different *E*_GABA_ values might be explained by nonhomogeneous subcellular distribution of chloride transporters. To test this hypothesis, we applied a dual approach consisting of immunohistochemical labeling of the K-Cl cotransporter KCC2 (Fig. 2g−i; Supplementary Fig. 3a) and the measurement of *E*_GABA_ at GC and PVI dendrites upon blocking KCC2 with the antagonist VU0240551 (10 µM; Supplementary Fig. 3b). Membrane-bound KCC2 immunolabelling was prominent at both soma and proximal dendrites of mature calbindin-expressing GCs with a decline towards distal sites (proximal: 2.5 ± 0.1 vs. distal: 1.8 ± 0.06 normalized fluorescence intensity; 41 and 39 dendritic sections; *p* < 0.001, two-way ANOVA test with Holm−Sidak pairwise comparison; Fig. 2g−i). In contrast, intensity of KCC2 antibody labeling in PVIs was ~2-fold higher at distal than proximal dendrites (distal: 3.4 ± 0.2 vs. proximal: 1.5 ± 0.2 normalized fluorescence intensity, 13 and 16 dendritic sections; *p* < 0.001), suggesting that different chloride regulation mechanisms between dendritic compartment may underlie the observed *E*_GABA_ gradient. Similar data were observed for adult rats (P71; Supplementary Fig. 3a). Moreover, blocking KCC2 resulted in a significant shift of *E*_GABA_ to more depolarized values in GCs (proximal *E*_GABA_: −60 ± 1.8, distal *E*_GABA_: −60.4 ± 3 mV; five GCs; *p* = 0.0005 and 0.008 respectively, two-tailed unpaired *t* test; Supplementary Fig. 3b), and a decline in the *E*_GABA_ gradient between proximal and distal dendritic sites in PVIs (proximal *E*_GABA_: −61.5 ± 4, distal *E*_GABA_: −67.6 ± 3.8 mV; five PVIs; *p* = 0.3, two-tailed paired *t* test; Supplementary Fig. 3b).

Thus, *E*_GABA_ is nonuniform across the somato-dendritic axis of PVIs and GCs supporting hyperpolarizing actions of GABA_A_R-mediated signals evoked at distal dendrites in both neuron types but divergent effects at proximal dendrites; a shunt or depolarization in PVIs but hyperpolarization in GCs.

### Dendritic inhibitory effects depend on excitatory strength

**Fig. 3:**
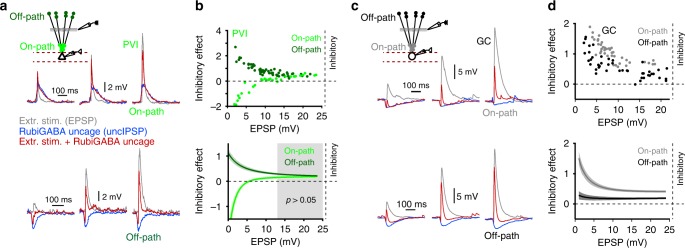
Inhibitory efficiency differentially depends on excitatory drive onto PVIs and GCs. **a**, **c** Inset, schematic illustration of the experimental design. Subthreshold EPSPs were evoked by extracellular stimulation (extr. stim; 0.1–0.2 ms) of the perforant path on the level of the middle molecular layer (gray lines) at a distance of ~150 µm to the recorded soma of PVIs (**a**) and GCs (**c**). IPSPs were evoked by sequential RubiGABA uncaging at seven randomly chosen proximal on-path (PVI bright green, GC gray filled circles) at the level of the inner molecular layer (25–75 µm distance to soma), or seven distal off-path (PVI dark green, GC black filled circles) spots in the outer molecular layer (200–250 µm distance to soma) distributed at apical dendrites of PVIs and GCs (0.5 ms uncaging duration, 2 ms inter-pulse interval between individual seven uncaging locations), prior to extracellular stimulation. Superimposed traces are individual EPSPs during control conditions (gray), uncIPSPs (blue) and PSPs evoked by extracellular stimulation and RubiGABA uncaging (red) at three extracellular stimulation intensities shown from left to right for on-path (upper set of traces) and off-path inhibition (lower set of traces). **b**, **d** Inhibitory effect of RubiGABA uncaging was plotted against EPSP amplitudes for on- and off-path inhibition in PVIs (**b**) and GCs (**d**). Upper graphs depict representative results from individual cells. Lower graphs summarize data from all experiments (14 PVIs, 19 GCs). Data were fitted with a single exponential function (Methods). Note, on-path inhibition was significantly more efficient across EPSP amplitudes in nongray areas for PVIs and for all EPSP amplitudes for GCs than off-path inhibition (*p* ≤ 0.05). Circles represent individual data points, lines with shades represent mean ± s.e.m. Gray square in (**b**) depicts area of no significant difference between on- and off-path inhibitory effects (*p* ≥ 0.05).

PVIs and GCs require convergent and synchronous excitatory inputs for their recruitment (PVIs: 15–60 synaptic contacts corresponding to 5–20 principal cells converging on one PVI^51^; GCs: ~55 contacts^23^). Therefore, in dependence on network activity, EPSP amplitudes will strongly fluctuate^29^. We examined the efficiency of on- and off-path-mediated GABAergic inputs in dependence on excitatory signal size, by evoking EPSPs of varying amplitude (1.1–25 mV) by extracellular stimulation of the middle molecular layer (Fig. 3) and uncaging RubiGABA at seven on- or off-path locations relative to the extracellular stimulation site. Uncaging sites were distributed over different apical dendritic branches (Fig. 3a, c, inset) to reproduce the broad distribution of synaptic contacts arising from single dendritic-inhibitory interneurons^38^. In PVIs, on-path GABAergic signals boosted EPSPs with amplitudes <5 mV but diminished EPSPs >5 mV (Fig. 3b, bright green), thereby homogenizing EPSP sizes (Supplementary Fig. 4). Off-path-mediated inhibitory efficiency exponentially declined with increasing EPSP amplitude (Fig. 3b, dark green) and converged towards inhibitory efficiencies equal to on-path inhibition for large EPSPs (≥13 mV; 14 PVIs; *p* > 0.05, two-tailed unpaired *t* test; Fig. 3b; gray area). A different picture emerged for GCs. Here, IE exponentially declined with EPSP amplitudes for both on- and off-path inhibition consistent with a predominantly hyperpolarizing action of GABAergic signals (Fig. 3c, d; gray vs. black, respectively, Supplementary Fig. 4). Moreover, on-path inhibition was over the entire range of tested EPSP amplitudes significantly more efficient than off-path inhibition (15 GCs; *p* < 0.05, two-tailed unpaired *t* test; Fig. 3d). A similar result was obtained if the extracellular stimulation site for activating glutamatergic inputs was positioned to the outer molecular layer in closer vicinity to RubiGABA uncaging locations (IE: 0.38 ± 0.05 vs. 0.03 ± 0.03 for distal excitation; eight GCs; *p* = 0.0005, two-tailed paired *t* test; Supplementary Fig. 5).

Taken together, with increasing strength of excitation, on- and off-path GABAergic inputs equalize their inhibitory efficiencies in PVIs, whereas on-path inhibition is over a wide range of EPSP amplitudes the more powerful inhibitory mechanism in GCs.

### Off-path inhibition efficiently controls recruitment of PVIs

**Fig. 4:**
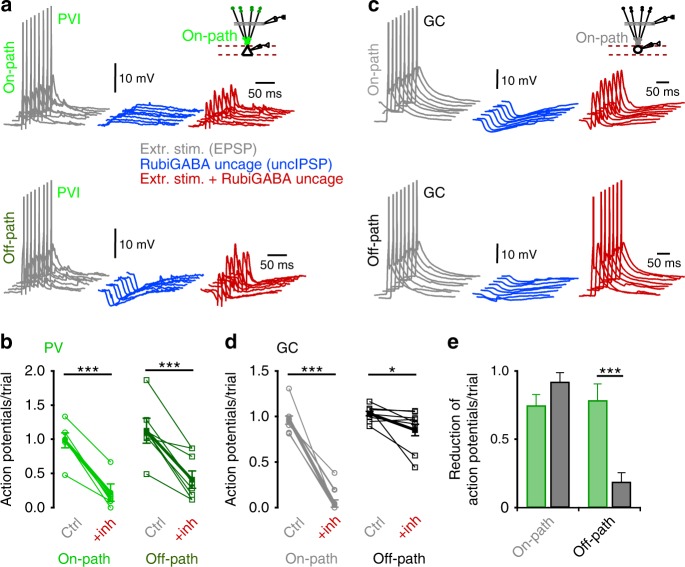
Off-path inhibition efficiently controls recruitment of PVIs but not GCs. **a**, **c** Experiments were performed as in Fig. 3, with supra-threshold perforant path fiber stimulation to evoke action potentials. UncIPSPs were induced by sequential RubiGABA uncaging at seven different distal or proximal dendritic locations. Gray traces show subsequently evoked action potentials under control conditions, blue traces represent individual uncIPSPs and red traces depict PSPs resulting from the interaction of uncIPSPs and supra-threshold EPSPs for on-path (upper traces) and off-path inhibition (lower traces). **b**, **d** Graphs summarize the effect of on- and off-path inhibition on action potential number per trial in PVIs (**b**) and GCs (**d**). Open circles and squares connected by lines represent data from individual cells; filled circles with lines and bars depict mean ± s.e.m. **e** Summary bar graph showing the average reduction in action potential number per trial during on- or off-path inhibition in PVIs (green) and GCs (gray). Note that on- and off-path inhibition are similarly effective in controlling action potential generation in PVIs but not in GCs. *p ≤ 0.05; ***p ≤ 0.001.

To test how the inhibitory efficiency of on- and off-path GABAergic inputs controls PVI and GC activity, we evoked supra-threshold excitatory signals by extracellular stimulation of the middle perforant path (action potential threshold −45.3 ± 2.31 and −46.0 ± 1.0 mV for PVIs and GCs, respectively; Fig. 4). In PVIs, distal off-path inhibition evoked by RubiGABA uncaging strongly reduced the probability of action potential generation, to a similar extent as on-path inhibition (from 1.01 ± 0.11 to 0.29 ± 0.11 and from 0.98 ± 0.07 to 0.22 ± 0.086 action potentials/trial, respectively; nine PVIs; *p* = 0.61, two-tailed Wilcoxon signed-ranks test; Fig. 4a, b). In contrast, on-path inhibition resulted in an almost full silencing of GCs, whereas off-path inhibition only mildly influenced their discharges (from 0.98 ± 0.04 to 0.04 ± 0.04 and from 0.94 ± 0.02 to 0.76 ± 0.06 action potentials/trial, respectively; *p* = 0.009, two-tailed Wilcoxon signed-ranks test; ten GCs; Fig. 4c, d).

Thus, on- and off-path inhibition are equally effective in silencing PVIs, indicating independence of the precise location of GABAergic inputs in controlling PVIs output. In contrast, on-path outperforms off-path inhibition in silencing GCs (Fig. 4e).

### GABA_A_R-conductances are enriched at distal PVI dendrites

**Fig. 5:**
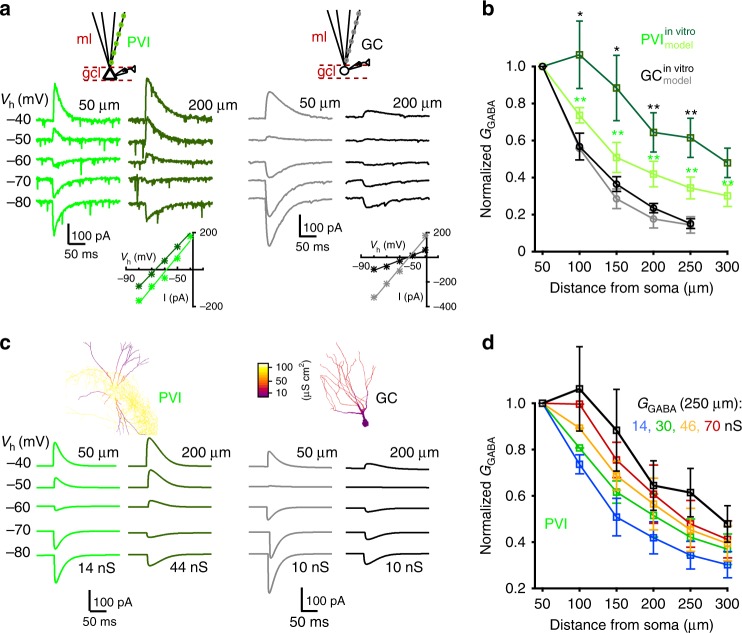
GABA_A_R-mediated conductances are enriched at distal PVI but not at GC dendrites. **a** Whole-cell voltage-clamp recordings from PVIs and GCs during RubiGABA uncaging at different positions along an apical dendrite (green squares). Holding potential (*V*_h_) was systematically changed to calculate the slope conductance underlying uncIPSCs (*G*_GABA_) from the resulting current−voltage relationship (lower insets). **c** PVI and GC single-cell models were used to evaluate the influence of signal attenuation on somatically measured *G*_GABA_. Insets, morphologies of representative PVI (left) and GC (right) used for single-cell computations. Color code represents membrane conductance distributions. GABAergic synapses were included along an apical dendrite (50 µm steps). Traces show somatic IPSPs evoked at somato-dendritic distances of 50 and 200 µm at various *V*_h_ in model PVIs (left) and GCs (right). *G*_GABA_ was determined from current−voltage relationships as in experiments (**a**), with a linear somato-dendritic *E*_GABA_ gradient constrained by in vitro results (five PVI and five GC model cells; Supplementary Fig. 6b). **b** Experimentally obtained *G*_GABA_ was normalized to the most proximal uncaging site (50 µm) and plotted as a function of somatic distance (dark green and black lines, six PVIs and five GCs, respectively). Experiments were reproduced in PVI (bright green) and GC (gray) models with uniform somato-dendritic *G*_GABA_ (14 and 10 nS for PVIs and GCs, respectively). Note, GC models closely reproduce the attenuation of inhibitory signals observed in vitro but not PVI models. **d** Plot as in (**b**) for PVI models with different somato-dendritic *G*_GABA_ distributions. Black line depicts in vitro data, blue line defines models with uniform *G*_GABA_ distribution. Green, yellow and red traces depict results from models with different linearly increasing *G*_GABA_ gradients (numbers refer to *G*_GABA_ at 250 µm, while at 50 µm the 14 nS conductance was kept constant). Note, a linear increase of *G*_GABA_ from 14 nS at 50 µm to >46 nS at 250 µm was the best fit to our in vitro data (yellow, red). Circles and squares with connecting lines depict mean ± s.e.m. *p ≤ 0.05; **p ≤ 0.01. Green asterisk refers to comparisons between in vitro and model PVI data; black asterisk refers to comparisons between PVIs and GCs in vitro.

What underlies the stronger distal inhibitory efficiency in PVIs compared to GCs? The opposing gradients in *E*_GABA_ between PVIs and GCs may be one important mechanism (Fig. 2); however, the dendritic distribution of *G*_GABA_ may also play a role. Indeed, electron-microscopical studies showed that GABAergic synapses are more numerous at distal CA1-PVI dendrites^52^. We therefore aimed to determine *G*_GABA_ along the somato-dendritic axis of PVIs and GCs by uncaging RubiGABA at dendrites with defined distances to the soma (50 µm steps; Fig. 5a, inset). UncIPSCs were recorded at varying holding potentials and *G*_GABA_ was derived from the steepness of the resulting current−voltage relationship (Fig. 5a). *G*_GABA_ monotonically declined as the uncaging location was moved away from the soma for both cell types; however, the decline was less pronounced in PVIs compared to GCs (Fig. 5b, dark green vs. black; Supplementary Fig. 6a). Indeed, at a somato-dendritic distance of 250 µm, the normalized *G*_GABA_ was ~4-fold higher in PVIs than in GCs (0.61 ± 0.1 vs. 0.15 ± 0.03 for six PVIs and six GCs respectively; *p* = 0.0036, two-tailed unpaired *t* test; Fig. 5b), suggesting different GABA_A_R distributions across the somato-dendritic axis.

Dissimilarities in dendritic architecture and passive cable properties can induce cell-type-specific attenuations of distally evoked signals^21–23,53^. To evaluate effects of dendritic filtering on the measured *G*_GABA_, we studied the propagation of inhibitory signals from their induction site to the soma using morphologically detailed computational models of PVIs and GCs^21–23,54,55^. Single-cell models were equipped with inverse somato-dendritic gradients in *R*_m_ as previously described (10–100 and 80–25 kΩ cm^−2^ from proximal to distal in PVIs and GCs, respectively) and with *E*_GABA_ values that linearly changed across the somato-dendritic axis, constrained by our experimental data (Supplementary Fig. 6b). Inhibitory conductances were included along apical PVI and GC dendrites with constant values of 14 and 10 nS, respectively. These values represented *G*_GABA_ estimates underlying uncIPSCs evoked at 50 µm distance from the soma of the two cell types. We replicated our in vitro experiments and used the slope of current−voltage relationships recorded at the soma of PVI and GC models to determine *G*_GABA_ (Fig. 5b, c). In GC models the attenuation of dendritic IPSCs was similar to the one observed in vitro, indicating that the apparent decay in the activated *G*_GABA_ predominantly arose from dendritic filtering (Fig. 5b, gray vs. black; five GC models). In contrast, PVI models showed significantly stronger IPSC attenuation than the experimentally evoked uncIPSCs (Fig. 5b, dark vs. bright-green; e.g. model PVI normalized *G*_GABA_ at 250 µm distance 0.36 ± 0.03 vs. in vitro PVI 0.61 ± 0.1; five PVI models and six PVIs; *p* = 0.006, two-tailed Wilcoxon rank sum test), suggesting that dendritic filtering might be balanced by enriched *G*_GABA_ at distal apical dendrites. Indeed, PVI models closely reproduced experimentally observed attenuations of dendritically evoked uncIPSCs under conditions of a linearly increasing somato-dendritic gradient of *G*_GABA_ from 14 nS at the soma to ≥46 nS at distal dendrites (Fig. 5d, in vitro black vs. model red and orange). This effect could not be reproduced by only changing the gradient of the somato-dendritic *R*_m_ (Supplementary Fig. 7) or the axial resistance (*R*_a_; Methods), supporting our conclusion that *G*_GABA_ gradually increases along apical dendrites of PVIs, while it remains uniform at the somato-dendritic axis of GCs.

### *E*_GABA_ and *G*_GABA_ shape efficiency of dendritic inhibition

**Fig. 6:**
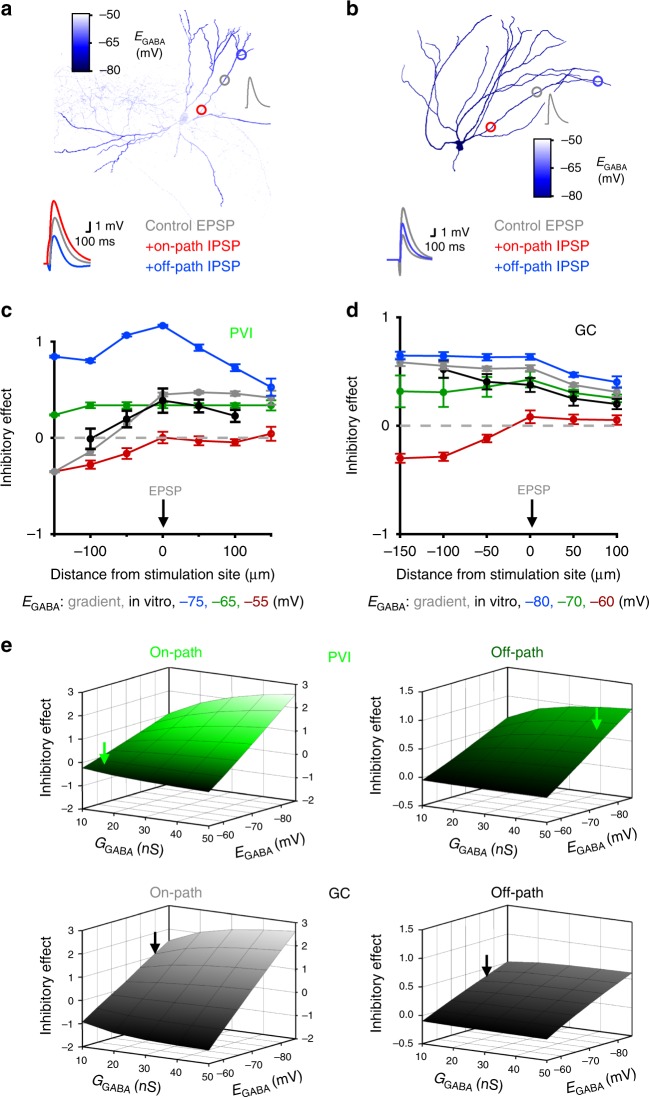
*E*_GABA_ and *G*_GABA_ gradients support the differential effect of on- and off-path inhibition. **a**, **b** Representative morphologically detailed PVI (left) and GC (right) models with experimentally constrained *E*_GABA_ gradients along the somato-dendritic axis (color scale represents *E*_GABA_). EPSPs were evoked at apical dendrites at a distance of 150 µm from the soma (5 nS, 5.1 ± 0.3 mV for PVIs, and 3 nS, 7.7 ± 0.6 mV for GCs, gray circles), while an inhibitory conductance was introduced at defined locations along the same dendrite (50 µm steps, red and blue circles represent *G*_GABA_ sites at 50 and 250 µm distance from the soma, respectively). Insets, superimposed representative traces of evoked EPSPs (gray), after induction of EPSPs with on- (red) or off-path inhibition (blue). **c**, **d** Inhibitory effect plotted as a function of distance from the stimulation site for PVI (**c**) and GC models (**d**) using different *E*_GABA_ distributions (three PVI and three GC models). Black lines represent experimental data (from Fig. 1e), gray lines depict results using a linear experimentally constrained *E*_GABA_ gradient, while blue, green and red traces represent models with uniform *E*_GABA_ values. **e** Inhibitory effect plotted against *G*_GABA_ and *E*_GABA_ for on- (left, 50 µm from soma) and off-path inhibition (right, 250 µm from soma) for PVIs (upper panels, three model cells) and GCs (lower panels, four model cells). Arrows point to experimentally (in vitro) obtained *E*_GABA_ and approximated *G*_GABA_ values for on- and off-path inhibition (see Figs. 2, 5).

To examine if the observed *E*_GABA_ and *G*_GABA_ distributions jointly account for the different dendritic inhibitory efficiencies in PVIs and GCs, we used model cells equipped with realistic *R*_m_ gradients and voltage-dependent conductances (Fig. 6). To reproduce experimental µEPSPs in PVIs, model cells were equipped with one excitatory conductance placed at an apical dendrite (5 nS, 150 µm distance to soma, 5.1 ± 0.3 mV) and introduced GABA_A_R-mediated signals at various locations along the dendrite (Fig. 6a). When inhibitory conductances had *E*_GABA_ values following our experimentally constrained linear gradient, the in vitro inhibitory efficiencies for both on- and off-path GABAergic signals were reproduced (Fig. 6c, gray vs. black, respectively). However, if *E*_GABA_ was set to more positive or more negative constant values (−55, −65 or −75 mV), the impact of inhibition diverged from the experimentally determined IE (Fig. 6c). Similarly, GC models equipped with a linear in vitro-based *E*_GABA_ gradient (Fig. 6b) or a constant *E*_GABA_ of −80 mV reproduced the experimentally obtained monotonic decline in inhibitory efficiency along the somato-dendritic axis of GCs (Fig. 6d, gray, blue and black, respectively), while deviations to more positive *E*_GABA_ values (−60 or −70 mV) resulted in different IE spatial profiles (Fig. 6d, red and green vs. black). Additionally, deviations from the realistic *E*_GABA_ markedly altered the dependency of inhibitory efficiency with EPSP size for on- and off-path inhibition in both cell types (Supplementary Fig. 8). Thus, *E*_GABA_ distributions along the somato-dendritic axis strongly influence the relationship between location of inhibition and its effect on EPSP size for both PVIs and GCs.

Finally, we systematically varied both factors *E*_GABA_ and *G*_GABA_ (Fig. 6e). Our analysis revealed that *E*_GABA_ and *G*_GABA_ collectively add to the effect of on- and off-path inhibition in both cell types; however, the impact of *E*_GABA_ was more prominent (Fig. 6e). This result was only negligibly influenced by the somato-dendritic *R*_m_ gradient in PVIs (Supplementary Fig. 9). For on-path inhibition, the dependence of inhibitory efficiency on *E*_GABA_ was less steep in PVIs compared to GCs indicating that *E*_GABA_ has a stronger influence on proximal inhibitory efficiency in GCs than in PVIs. The opposite was the case for off-path inhibition; its inhibitory efficiency depended more on *E*_GABA_ in PVIs than in GCs (Fig. 6e).

### Off-path inhibition is optimized for controlling PVIs output

**Fig. 7:**
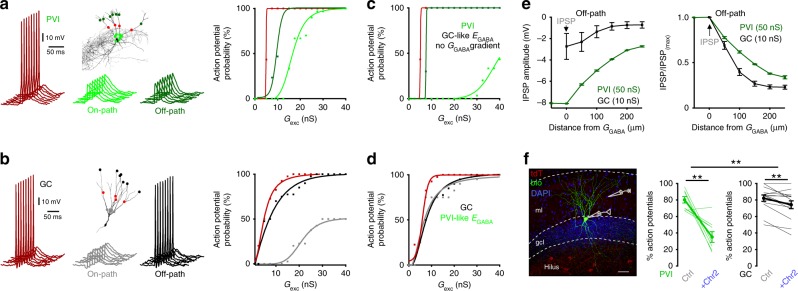
Off-path inhibition controls action potential generation in PVI but not in GC models. **a**, **b** Action potentials in PVI and GC models were induced by excitatory conductances (*G*_exc_) activated at apical dendrites at 150 µm distance to the soma (red circles) reproducing in vitro experiments (Fig. 4a, c). GABA_A_R-mediated conductances (*G*_GABA_) were initiated at seven dendritic sites located either on- or off-path in relation to the *G*_exc_ site (14 and 54 nS in three PVI models, 7.5 and 7.5 nS in four GC models, for on- and off-path inhibition, respectively). Left traces, subsequently evoked individual action potentials (red) in a PVI (upper) and a GC (lower). Middle, voltage traces resulting from the interaction between excitatory signals and on-path inhibition in PVI (upper, bright green) and GC models (lower, gray). Right traces, same as middle but for off-path inhibition. Right graphs, probability of action potential generation plotted as a function of *G*_exc_ without inhibition (red) and in presence of on-path (PVIs, light green; GCs, gray) or off-path inhibition (PVIs, dark green; GCs, black). Data were fit with a sigmoid function. **c**, **d** Discharge probability was plotted against *G*_exc_ for PVIs (upper), equipped with GC-like *E*_GABA_ and with constant *G*_GABA_, and for GCs (**d**), equipped with a PVI-like *E*_GABA_ gradient, in control conditions (red) and during on- (gray) or off-path (black) inhibition. **e** Left, an inhibitory input was placed at a distance of 250 µm from the soma in PVI and GC models. IPSP amplitude along the dendrite is plotted against distance from its induction site (arrow). Right, IPSP amplitudes at different dendritic locations were normalized to the maximum amplitude at their induction site and plotted against distance from the *G*_GABA_ location. **f** Left, representative confocal stack of a biocytin-filled PVI in an acute slice from a SOM-Cre mouse expressing channelrhodopsin-2 (Chr2) and tdTomato (tdT) after viral injection (scale bar 50 µm). Single action potentials were evoked by extracellular perforant path stimulation and the effect of a short overlapping Chr2 pulse on action potential probability is plotted for PVIs and GCs. Squares and circles represent means and lines depict ±s.e.m. Thin lines in panel (**f**) represent individual experiments. **p ≤ 0.01.

How do the different properties of GABAergic synaptic transmission in PVIs and GCs impact action potential generation? To address this question we used active single-cell PVI and GC models equipped with the experimentally defined *E*_GABA_ and *G*_GABA_ gradients (Fig. 7; Methods). In PVI-models, on- and off-path inhibition prevented spike induction, similar to in vitro phenotypes (Fig. 7a vs. Fig. 4a) but silencing could be overcome by further increasing the synaptic excitatory conductance (*G*_exc_; Fig. 7a, right). In GCs-models, similar to our in vitro data, on-path inhibition in the range of unitary PVI-GC conductances^34,56^ (7.5 nS) was an efficient silencing mechanism (Fig. 7b vs. Fig. 4c) which could not be counteracted by increasing *G*_exc_ (Fig. 7b, right). In contrast, off-path inhibition had only a mild effect on spike generation even when excitatory inputs were moved closer to the GABAergic distal conductance site (Supplementary Fig. 10a, b). Moreover, when PVIs were equipped with a constant GC-like hyperpolarizing *E*_GABA_ value, distal inhibition was no longer efficient in controlling action potential generation (Fig. 7c, bright green), while GCs equipped with a PVI-like *E*_GABA_ gradient showed a milder silencing effect of proximal inhibition (Fig. 7d, gray). When IPSP propagation along dendrites was measured, we observed that distally evoked IPSPs in PVIs were larger across the entire somato-dendritic axis compared to GCs (i.e. IPSP amplitude −3.9 ± 0.01 and −0.98 ± 0.07 mV at a distance of 150 µm in three PVI and five GC models from the IPSP induction site, respectively; *p* < 0.0001, two-way ANOVA with Holm−Sidak analysis; Fig. 7e, left). Moreover, attenuation was milder in PVI than in GC models (i.e. IPSP attenuated to 33.8 ± 2% and to 23 ± 2% from the induction site at 250 µm from the soma in three PVI and five GC models, respectively; *p* < 0.001, two-way ANOVA with Holm−Sidak analysis; Fig. 7e, right). This finding was independent on the kinetic properties of unitary *G*_GABA_ used in PVIs and GCs (decay τ 2 and 4 ms, respectively;^43^ Supplementary Fig. 10c). Thus, we conclude that the particular *E*_GABA_ and *G*_GABA_ dendritic distributions together with the mild IPSP attenuation along apical dendrites in PVIs can support the high efficiency of distal inhibition in controlling PVI output.

To test this conclusion at physiological conditions, we analyzed the effects of distal dendritic inhibition provided by SOMIs on the recruitment of PVIs and GCs. We injected Cre-inducible adeno-associated viruses encoding channelrhodopsin-2 and tdTomato into the dentate gyrus of adult SOM-Cre mice (Fig. 7f, left). We stimulated the middle molecular layer to evoke action potentials in PVIs and GCs, and tested the effect of ChR2-mediated recruitment of SOMI-axons in the outer molecular layer on their discharge probability. As in our RubiGABA uncaging experiments, synaptic inhibition at distal PVI dendrites induced larger reductions in discharge probability than in GCs (PVIs: from 80.3 ± 3.9 to 35.1 ± 6.5%; GCs: from 82.3 ± 3.9 to 74.2 ± 4.7%; 9 PVIs and 14 GCs; *p* = 0.002 and 0.003, respectively, two-tailed paired *t* test; PVI vs. GC effect: *p* = 0.002, two-tailed Wilcoxon rank sum test; Fig. 7f, right).

### Narrow time window for dendritic inhibitory efficiency in PVIs

**Fig. 8:**
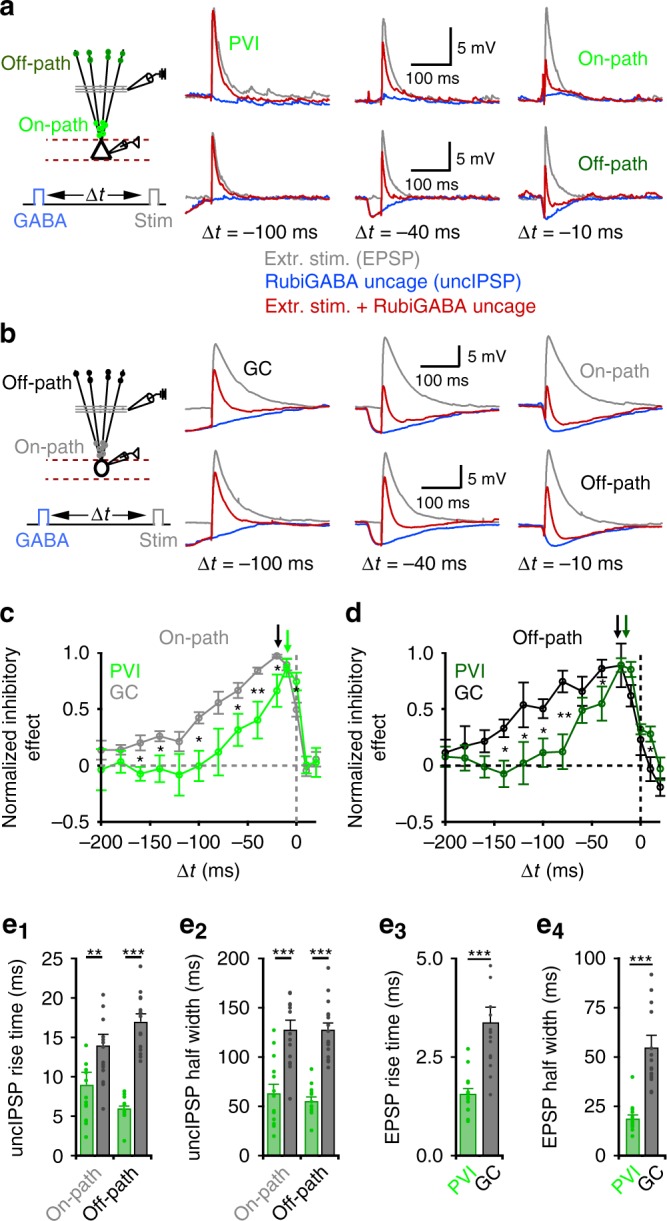
Inhibition is more temporally precise in PVIs than in GCs. **a**, **b** Left, schematic illustration of the experimental design. EPSPs were evoked by extracellular stimulation of the middle molecular layer (~150 µm distance from the soma) and recorded at the soma of PVIs (**a**) or GCs (**b**) in the absence or presence of dendritic inhibition. IPSPs were evoked by sequential RubiGABA uncaging (uncIPSPs) at seven dendritic positions randomly distributed on- (25–75 µm distances to soma) or off-path (>200 µm distance from the soma). EPSP amplitude was kept constant while the time window (∆*t*) between the onset of RubiGABA uncaging and extracellular stimulation was systematically changed between −200 and 20 ms. Right, superimposed representative traces of control EPSPs (gray), uncIPSPs (blue) and EPSPs in presence of uncIPSCs (red) for on-path (upper traces) and off-path (lower traces) inhibition at three different ∆*t*. **c**, **d** Summary plots show inhibitory effects of on-path (**c**) and off-path (**d**) inhibition for PVIs (bright and dark green, respectively; eight PVIs) and GCs (gray and black, respectively; 11 GCs) as a function of ∆*t*. Note, maximal inhibitory effects for on- and off-path inhibition were achieved at ~10 ms ∆*t* in PVIs and at ~20 ms ∆*t* in GCs. **e**_**1–4**_ Bar graphs summarize functional properties of synaptically evoked EPSPs and uncIPSPs. Note, signals are faster in PVIs (green) compared to GCs (gray). Bars with lines and circles with lines represent mean ± s.e.m. *p ≤ 0.05; **p ≤ 0.01; ***p ≤ 0.001.

Timing of inhibition in relation to excitation has an important influence on inhibitory efficiency^57^. To analyze time-dependencies of dendritic inhibition, we induced EPSPs in PVIs and GCs by extracellular stimulation of the perforant path (*t* = 0 ms) and systematically varied the latency of on- or off-path inhibitory signals evoked by RubiGABA uncaging (Δ*t*: −200 to 20 ms) relative to EPSP onset (Fig. 8). In PVIs, on- and off-path inhibition were most effective if induced ~10 ms prior to EPSP onset (Fig. 8a, c, d; arrows). Deviations from this optimal Δ*t* resulted in a sharp decline in IE (Fig. 8c, d, green). In GCs, on- and off-path GABAergic signals were most effective if induced ~20 ms prior to EPSP onset (Fig. 8b−d; arrows). A systematic change in Δ*t* resulted in a decrease in IE; however, this decline was less steep than in PVIs, resulting in a two- to three-fold broader time window of half-maximal inhibition in GCs (Fig. 8c, d, gray and black).

Time windows of signal integration are largely defined by kinetic properties of excitatory and inhibitory signals. Somatically recorded uncIPSPs in PVIs had, independent of their induction site, faster time courses than those in GCs (rise time: PVI 8.5 ± 1.6 ms and 5.6 ± 0.3 ms vs. GC 14.2 ± 1.4 ms and 17.2 ± 1 ms for on- and off-path; *p* = 0.006 and 3 × 10^−6^, respectively; Fig. 8e_1_; half-duration: PVI 63.4 ± 8.9 and 55.4 ± 4.3 ms vs. GC 128.3 ± 9.7 and 128.0 ± 6.7 ms for on- and off-path; 15 PVIs and 17 GCs; *p* = 0.0003 and 3.75 × 10^−6^ respectively, two-tailed Wilcoxon rank sum test; Fig. 8e_2_). Similarly, EPSPs evoked by perforant path-stimulation were faster in PVIs compared to GCs (rise time: PVI 1.6 ± 0.1 ms vs. GC 3.4 ± 0.4 ms; *p* = 1.8 × 10^−5^; Fig. 8e_3_; half-duration: PVI 18.9 ± 1.7 ms vs. GC 57.9 ± 3.9 ms; *p* = 3.1 × 10^−6^, two-tailed Wilcoxon rank sum test; Fig. 8e_4_). Interestingly, in PVIs off-path-mediated inhibition tended to be faster than on-path inhibition (Fig. 8e_1,2_), which can be attributed to fast distal dendritic charge distribution^22^ or different GABA_A_Rs subunit compositions.

Thus, the faster time course of glutamatergic and GABAergic signals at PVI dendrites define a narrow time window for input integration and a high inhibitory efficiency independent of the dendritic induction site. In contrast, the slower time course of synaptic signals in GCs supports input integration and the high efficiency of proximal inhibition.

## Discussion

This study provides new information on the role of dendritic GABA_A_R-mediated signaling in controlling excitability of dentate gyrus PVIs and GCs. We show that in PVIs, *E*_GABA_ of proximal GABAergic signals resides between resting membrane potential and action potential threshold, but it is below the resting potential for distally evoked GABAergic signals. Moreover, the density of GABA_A_Rs is lower at proximal than distal dendrites. The combination of proximal shunting and strong distal hyperpolarizing inhibition in PVIs introduces functional advantages to the dentate gyrus network. In dependence on excitatory input strength, proximal GABAergic shunting can boost small but diminish large EPSPs, thereby resulting in a homogenization of excitatory signals, a mechanism proposed to improve spike timing in PVIs and synchrony of network oscillations^49^. With increasing excitatory signal strength, proximal and distal inhibition equalize their inhibitory effects, thereby making the impact of GABAergic signals on PVIs’ output less dependent on its precise dendritic location. In contrast, distal GABA_A_R-mediated signaling in GCs has only mild influences on their excitability and activity. This finding markedly differs to observations in CA1 and cortical layer five pyramidal cells where distal dendritic inhibition effectively controls action potential generation^11,16,47^. Our data indicate a constant somato-dendritic *G*_GABA_ gradient and strong attenuation of distally evoked signals, rendering proximal inhibition as the dominant mechanism controlling GCs’ excitability.

In vivo and in vitro investigations revealed that hippocampal fast-spiking interneurons upon recruitment by glutamatergic inputs generate action potentials at high temporal precision^8,29,58,59^, a property proposed to be of key importance for synchronizing principal cell assemblies^43,60–62^. Moreover, neuronal network computations revealed that shunting perisomatic inhibition in fast-spiking interneurons improves synchrony of fast network oscillations^49,62^. Besides the important roles of perisomatic inhibition, our data show that distal dendritic inhibition also has a powerful influence on perforant path-mediated PVI recruitment (Fig. 4). However, how can this powerful control in PVIs’ excitability by dendritic inhibition be explained? PVIs display unique membrane characteristics and synaptic input properties in comparison to principal cells^3–5^ and other types of GABAergic interneurons^20,63^. Apical dendrites of dentate gyrus-PVIs neither generate dendritic spikes^24^ nor undergo synaptic plasticity^44^. They express low levels of voltage-gated Na^+^ and high levels of voltage-gated K^+^ channels^24,64^, which confer linear summation mechanisms of excitatory signals and support the integration of spatially distributed inputs. PVIs possess a nonuniform *R*_m_ along the somato-dendritic axis with lowest values close to the soma (*R*_m_ ~ 10 kΩ cm^2^, <120 µm) and increasing values to distal dendrites^22^ (>120 µm, *R*_m_ ~ 100 kΩ cm^−2^). The high distal *R*_m_ will boost the amplitude of local, synaptically evoked EPSPs and together with their low attenuation during dendritic propagation^22,24^ enhance their ability to induce somatic action potentials. In the case of inhibitory signals, our work shows that the combination of hyperpolarizing *E*_GABA_ together with a high *R*_m_^22^ and a high distal *G*_GABA_ supports strong GABAergic IPSPs at outer dendritic compartments. Our immunohistochemical data indicate that *E*_GABA_ gradients may be supported by different KCC2 densities across the somatodendritic axis of PVIs (Fig. 2g−i). The observed *G*_GABA_ gradients may be explained by a higher number of GABAergic synapses at distal dendrites as observed in CA1-PVIs^52^ or by different GABA_A_R subunit compositions with specific gating kinetics^65^. Thus, the resulting strong distally evoked IPSPs together with their mild attenuation can circumvent somatic action potential generation. Finally, fast time courses of EPSP and IPSPs narrow their temporal interaction (Fig. 8^24,51,64,66^), indicating that high inhibitory efficiency can be particularly achieved when synaptic inputs are synchronized, such as during fast network oscillations^10,43,49^. Thus, PVIs are capable of sampling inhibitory and excitatory signals across the whole dendritic tree in a spatially unspecific manner.

Our results imply a different functional role of distal dendritic inhibition in GCs, which has only a mild effect on GCs output and a lower influence on the amplitude of dendritically evoked EPSPs compared to PVIs (IE: 0.18 vs. 0.34 for 10 mV EPSPs, respectively). A small IE was also observed when excitatory inputs were close to GABAergic input sites (Supplementary Figs. 4, 10). Although our experiments using iontophoretic stimulation and one-photon RubiGABA uncaging will activate both post- and extrasynaptic GABA_A_Rs, our results are in agreement with studies recruiting specifically postsynaptic GABA_A_Rs using pair recordings^18^ or optogenetics^17,46^, showing a compartmentalized effect of dendritic inhibition on principal cell signaling.

What mechanisms underlie the low impact of off-path inhibition in GCs? Similar to PVIs, GC dendrites express low levels of voltage-gated ion channels which confer linear integration of synaptic potentials^21,23^ (but see ref. ^67^). The somato-dendritic *R*_m_ gradient is opposite to the one in PVIs^54^. It is two-fold lower at distal dendrites than in PVIs resulting in smaller local EPSPs^21,23^. Moreover, dendritically evoked EPSPs undergo a stronger attenuation during their propagation to the soma^21,23^ (~4.5-fold larger at somato-dendritic distance of ~300 µm^22,23^). We provide evidence that GABA_A_R-mediated signal properties contribute to the low efficiency of distal inhibition. A nonuniform *E*_GABA_ gradient at the somato-dendritic axis causes a stronger hyperpolarization at proximal than distal sites and acts synergistically with the uniform *G*_GABA_ in generating smaller IPSPs at distal compared to proximal dendrites. Distally induced IPSPs are stronger attenuated during their propagation to the soma compared to PVIs (1.4-fold higher attenuation at 150 µm distance; Fig. 7e, f), and this attenuation will be even stronger for inputs located at spines^41^. Thus, the visibility of distal dendritic inhibition is low across the somato-dendritic axis. What might be the functional relevance of distal dendritic inhibition in GCs? Previous studies indicate that it restricts local dendritic Ca^2+^ transients^17,18,46^, which are necessary for promoting long-lasting synaptic plasticity at GCs as well as CA1 pyramidal cell dendrites^47,67–69^. This hypothesis fits to the previously shown enhanced synaptic plasticity-dependent cfos expression^70^ in GCs upon SOMI silencing^40^ and to the substantial proportion of GABAergic synapses (~55%) targeting GC spines in the outer molecular layer^41^, which largely originate from SOMIs^37^. Thus, distal inhibition may locally modulate dendritic synaptic signaling and plasticity.

In summary, our results indicate that PVIs and GCs show fundamentally different dendritic integration mechanisms. During low levels of perforant path-mediated excitatory drive to the dentate gyrus, such as during novel contextual exposure^31,71^, shunting proximal inhibition will boost excitatory signals in PVIs and thereby support a basal PVI-mediated perisomatic inhibitory output onto GC populations. During this condition some GCs may overcome proximal inhibition and form new synchronously active cell associations. Repetitive exposure to the same environment will increase excitation levels^31,71^ and enhanced dendritic inhibition, which will in turn reduce PVI recruitment and modulate synaptic plasticity at GC distal dendrites. Such a mechanism may enable PVIs to provide phasic proximal inhibition onto GC populations balanced to the network activity state and allow subpopulations of GCs to form new coalitions representing new contextual information.

## Methods

### Slice preparation and electrophysiology

All experimental procedures were performed in accordance to national and institutional legislations (licenses X-13/03S, X-16/306S and G-15/106 approved by the Regierungspräsidium Freiburg). Transverse acute hippocampal slices (300–350 µm, Leica VT1200 vibratome) were obtained from Wistar rats (P19-28) of either sex. Slice preparation and whole-cell patch-clamp recordings^55^ were performed using ACSF containing (in mM): 125 NaCl, 25 NaHCO_3_, 2.5 KCl, 1.25 NaH_2_PO_4_, 25 d-glucose, 2 CaCl_2_ and 1 MgCl_2_ (oxygenated with 95% O_2_/5% CO_2_) supplemented with 2 µM CGP55845 to block GABA_B_ receptors. Whole-cell recordings (at 30–34 °C) were done using glass pipettes of 2–5 MΩ when filled with a solution containing (in mM): K-Gluconate 140, 132 or 127 for solutions with a calculated chloride equilibrium potential of −72.7, −63.3 or −54.2 mV respectively, KCl 4, 8 or 13, HEPES 10, MgCl_2_ 2, Na_2_ATP 2, EGTA 10, 0.125 Alexa Fluor-488 and 0.15% biocytin (pH = 7.2; 290–310 mOsm). Whole-cell current-clamp recordings were obtained using an EPC 10 (HEKA) or a Multiclamp 700B (Molecular Devices) amplifier, filtered at 5 kHz and digitized at 40 kHz. Loaded recording pipettes had series resistances of 8–20 MΩ, which was compensated using the bridge balance in current-clamp mode and to 85% (time lag 5 µs) in voltage-clamp. For stimulus generation and data acquisition, we used custom-made programs (FPulse) written in Igor (Wavemetrics). Perforated-patch recordings were performed with an intracellular solution resulting in an equilibrium potential for chloride of −63.3 mV, supplemented with Gramicidin-A (Sigma) 50–60 µg/ml, freshly prepared from a DMSO stock. Recordings were performed in presence of AMPA and NMDA receptors blockers (20 µM CNQX and 100 µM D-APV, respectively) after series resistance fell below 150 MΩ. Integrity of the perforated-patch was continuously tested and confirmed by the absence of Alexa Fluor-488 somatic fluorescence (Fig. 2a). For measuring *G*_GABA_ activated by RubiGABA uncaging, whole-cell voltage-clamp recordings were performed using an intracellular solution that blocked voltage-dependent conductances (in mM): Cs-Gluconate 130, TEACl 8, QX-314 1, HEPES 10, MgCl_2_ 2, Na_2_ATP 2, EGTA 10, GTPNa_3_ 0.5 and 0.15 % biocytin (pH = 7.2; 303 mOsm). Extracellular stimulation was obtained using a glass pipette (~1 MΩ) filled with a HEPES-buffered Na^+^-rich solution. For current-clamp experiments in PVIs and GCs, membrane potentials were kept at ~−65 and ~−70 mV, respectively, by constant current injection, thereby reproducing their in vivo membrane resting potential^29^. On-path GABAergic signals in Fig. 2a were induced by positioning an extracellular stimulation pipette at the granule cell to molecular layer border and off-path inhibition was evoked by positioning the stimulation pipette in the outermost region of the molecular layer (0.1–0.2 ms pulse duration; 50 V). Subthreshold EPSP amplitudes were evoked by an extracellular stimulation pipette located in the middle molecular layer and randomly altering stimulus intensity (Fig. 3). IPSPs were recorded at different membrane holding potentials to determine *G*_GABA_ (Fig. 5). Trains of action potentials were evoked by 1-s-long current injections and discharge frequencies were determined as the reciprocal of inter-spike intervals. *R*_in_ was measured from the average current response (ten traces) at the end of a −10 mV, 1 s voltage pulse. Discharge probability was defined as the number of action potentials evoked by extracellular stimulation divided by the number of stimulus trials.

### Glutamate microiontophoresis and GABA uncaging

Local excitatory postsynaptic potentials were achieved using glutamate microiontophoresis^72^. Infra-red Dodt-contrast was used to visually identify cells and subsequently two-photon laser (Coherent, Chameleon Ultra II) scanning microscopy (Femto-Alba, Femtonics) was used to visualize Alexa Fluor-488-filled dendrites of PVIs and GCs using ×20 or ×40 objectives (water immersion, NA 1.0, Zeiss). For microiontophoresis, glass pipettes (20–80 MΩ) were filled with 150 mM l-glutamic acid (pH 7.0) and 50 µM Alexa Fluor-488. The pipette tip was approximated to dendrites at a distance of ~150 µm to the soma until an increase in spontaneous activity was observed, indicating close proximity to the recorded cell dendrite (<2 µm distance between pipette tip and dendritic surface). Glutamate was then kept in the pipette using constant positive current (1–5 nA) and expelled using 0.1–0.4 ms negative current pulses (1 µA) with a microiontophoresis system (MVCS-02, NPI Electronic). For GABA uncaging, slices were perfused with an extracellular solution containing RubiGABA (30 µM, prepared from a 1 mM stock dissolved in HEPES-buffered Na^+^-rich solution and kept at −80 °C) with a total volume of 8–15 ml (~32 °C). Experiments were performed in the dark to avoid unintentional uncaging of RubiGABA. Uncaging was induced using a 488 nm diode-pumped laser (Tectonics) coupled to the optical path of the two-photon laser and therefore positioned by the scanning mirrors and software of the Femtonics imaging system. To maintain stimulation intensity constant across dendritic compartments and cell types, duration (0.5 ms), laser power (0.5 mW) and RubiGABA concentration were kept constant throughout experiments and cell types. For experiments shown in Fig. 1, RubiGABA was uncaged at one dendritic position per trial with 4 ms preceding the onset of µGlut to compensate for the rise time of uncIPSPs. Distances between the location of µGlut stimulation and RubiGABA uncaging were measured online. To mimic activation of dendrite-targeting interneurons (Figs. 3, 4, 8), seven uncage positions were placed at different apical dendritic branches of the loaded cell at the level of the outer or the inner molecular layer, on- and off-path relative to the EPSP induction site, respectively (0.5 ms uncaging duration, 2 ms inter-pulse-interval between individual uncaging sites; uncaging series was initiated 20 ms prior to extracellular stimulation) and repeated every 15 s. For each cell, stimulated dendrites were located at similar depths in the slice (maximum *z*-axis distance between stimulated dendrites 35 µm). In this range of tissue depth, the amplitude of locally evoked IPSPs was not affected (Supplementary Fig. 2b). The effects of RubiGABA uncaging on spike generation were measured by using supra-threshold extracellular stimulation of the medial perforant path (Fig. 4). In these experiments, uncage laser intensity was set to a value that produced a clear reduction on action potential generation when directed to GC and PVI dendrites located in the inner molecular layer. Laser power was then kept constant for subsequent measures on the impact of RubiGABA uncaging on dendrites in the outer molecular layer of the same cells. GC experiments were excluded if RubiGABA uncaging resulted in IPSPs < 0.5 mV.

### Optophysiology

Recombinant adeno-associated viruses (rAAVs) encoding Channelrhodopsin-2 (Chr2) and tdTomato were injected into the ventral dentate gyrus (relative to bregma: *x*, 2.5 mm; *y*, 2.9 mm; *z*, 2.3, 2.6, 2.9 mm) of SOM-Cre mice (*Sst*^*tm2.1(cre)Zjh*^*/J*, Jackson Laboratory stock: 013044)^39^. The expression cassette of the rAAV contained tdTomato and Chr2 between two inverted loxP sites (rAAV1.CAGGS. Flex.Chr2. tdTomato.WPRES.SV40; Addgene catalog #18917). Slices were prepared >14 d after injection. Full-field illumination (488 nm, 20 ms; pE-100; CoolLED) was applied 5 ms before extracellular stimulation of the perforant path at an intensity just sufficient to evoke action potentials. Twenty to sixty trials with and without light-mediated SOMI-activation were used for calculating the probability of action potential generation.

### Neuronal staining and confocal imaging

After in vitro experiments, slices were fixed overnight in 4% paraformaldehyde and subsequently washed in 0.1 M phosphate-buffer (24 h) and 0.025 M phosphate-buffered solution (PBS) at room temperature. For KCC2 immunohistochemistry, brains were dissected and incubated in 4% paraformaldehyde overnight and 50 µm floating sections were obtained using a vibratome (Leica VT1000S). Slices were incubated with primary antibodies against PV (rabbit or mouse anti-parvalbumin, 1:1000 Swant), calbindin (guinea pig, 1:500, Synaptic Systems) or KCC2 (rabbit 1:500, Milipore) diluted in PBS containing 10% goat-serum and 0.3% triton X-100 for 24 h. Slices were afterwards incubated with Alexa Fluor-488 anti-mouse (1:1000, Jackson ImmunoResearch), Cy3-conjugated anti-rabbit (1:1000, Jackson ImmunoResearch), Alexa Fluor-647 anti-guinea pig or with streptavidin-conjugated Alexa Fluor-647 (for biocytin-filled cells, 1:1000, Jackson ImmunoResearch) for 4–24 h (4 °C). Slices were counterstained with DAPI (5 min) and mounted in Mowiol. Confocal image stacks of labeled neurons were obtained with a laser scanning confocal microscope (LSM-710, Zeiss) using ×40 or ×63 magnification objectives (Zeiss Achroplan). After in vitro whole-cell recordings using two-photon or confocal microscopy, 44 cells were identified as basket and 20 as axo-axonic cells based on their characteristic axonal distributions^36^.

### Single-cell models

Single-cell simulations were performed with NEURON 7.3, 7.5^73^ using detailed passive cable models previously developed on the basis of five morphologically reconstructed PVIs^22,55^ and five GCs^21^. For PVIs, *R*_a_ was set to 170 Ω cm and the specific membrane capacitance (*C*_m_)^22^ to 0.9 μF cm^−2^. *R*_m_ was nonuniformly distributed with somatic *R*_m_ set to 10 kΩ cm^2^ and the more distal tips of dendrites to 100 kΩ cm^2^ using a exponential somato-dendritic gradient. The exponential gradient in *R*_m_ had a constant (*τ*) of five, unless indicated, resulting in the best fit to the experimental data (Supplementary Fig. 7). To reproduce the fast-spiking nonaccommodating PVI phenotype, we used voltage-gated conductance densities (*g*) with somato-dendritic distributions following previous experimental and single-cell modeling data of dentate gyrus PVIs^22,24,74^. In PVIs, density of *g*_Na_ is high at the soma and declines with distance^24^ and was therefore set at the soma, axon and dendrites to 25, 25 and 1 mS cm^−2^ respectively. In Fig. 7, *g*_Na_ was set to 55, 85 and 0 mS cm^−2^ to reach action potential threshold values similar to those observed in vitro (d*V*/d*t* > 20 V s^−1^ = −41.8 ± 1.1 mV, 3 model PVIs). *g*_K_ and *g*_Ih_ were uniformly distributed with 20 mS cm^−2^ and 1 pS µm^−2^, respectively^24,55^ (Supplementary Table 1).

In GC models, *R*_a_ was set to 210 Ω cm and *C*_m_ to 1 μF cm^−2^ at the soma and primary dendrites, and was scaled to 1.6 μF cm^−2^ to compensate for the high density of spines at GC dendrites^21^. Changing *R*_a_ from 210 to 170 Ω cm ^23^ in GCs had no influence on the attenuation of IPSPs along the somato-dendritic domain (data not shown). *R*_m_ was nonuniform in GCs with 80 kΩ cm^2^ at the soma and perisomatic dendritic areas, and 50 kΩ cm^2^ at proximal and distal dendrites. We introduced a previously reported set of conductances that closely replicate the physiological phenotype of GCs^54^. Briefly, transient sodium channels (Na), L- T- and N-type calcium channels (CaL, CaT, CaN), fast and slow rectifiers (fK_DR_, sK_DR_), A-type potassium channels (A_K_), BK and SK calcium-dependent potassium channels (BK, SK), a slow after hyperpolarization conductance (sAHP) and a voltage-dependent cationic somatic current (*U*_c_) were implemented with heterogeneous distribution over the somatodendritic domain^54^ (available at https://senselab.med.yale.edu/ModelDB/showModel?model=169240&file=/DGC/DGC_Biophysics.hoc#tabs-2; Supplementary Table 2). As in our in vitro current-clamp experiments, the resting membrane potential was maintained at defined values by injecting current in the soma of PVI and GC models (PVIs: −65 mV, 40 to 65 pA; GCs: −70 mV, −5 to −31 pA).

For estimating the somato-dendritic distribution of *G*_GABA_ in PVI and GC models (Fig. 5), we compared our experimental data to models that replicated the specific recording conditions. We omitted all Na+ and K^+^ conductances from PVI and GC models and clamped the somatic potential using a simulated 10 MΩ pipette. *G*_exc_ and *G*_GABA_ inputs were added as point conductances using the netcon function. *G*_exc_ was simulated using the sum of two exponential functions with *τ*_rise_ = 0.1 ms and *τ*_decay_ = 8 ms and a reversal potential of 0 mV. Synaptic GABA_A_R-mediated conductances were simulated with a *τ*_rise_ and *τ*_decay_ of 0.1 and 20 ms, respectively. These kinetic parameters of *G*_exc_ and *G*_GABA_ were chosen to closely reproduce our in vitro experiments (Fig. 1). A subset of data was repeated using *τ*_decay_ of 2 and 4 ms for *G*_GABA_ in PVIs and GCs, respectively^43^. To compensate for the attenuation of propagating signals, we used PVI and GC passive cable models to determine the local *G*_GABA_ activated by RubiGABA uncaging at a perisomatic distance of 50 µm in vitro (PVI: 6.7 ± 0.7 and GC: 8.2 ± 1.7 nS measured in vitro; Supplementary Fig. 6a). We identified a local *G*_GABA_ of 14 and 10 nS in our PVI and GC models, respectively, which were used throughout simulations. These *G*_GABA_ values resemble perisomatic GABA_A_R-mediated conductances evoked at similar somato-dendritic distances from two converging basket cells (BCs) onto one GC or BC^34,56^.

For testing the effect of inhibition on action potential generation in PVI and GC models (Fig. 7), seven GABAergic inputs were distributed over different apical model dendrites, either on-path (~50–100 µm from the soma) or off-path (~200–250 µm from the soma), while excitatory inputs were inserted at individual dendrites at a distance of ~150 µm from the soma. Background activity was modeled as stochastic point-conductances at the soma with *G* values of 0.25 and 5 nS and reversal potentials of 0 and −70 mV, representing glutamatergic and GABAergic inputs, respectively^75^.

### Data analysis and statistics

Physiological and modeling data were analyzed using custom-made IgorPro analysis routines (Wavemetrics). Statistical analysis was performed using Excel (Microsoft) with “Real Statistics Excel Resource Pack” or SigmaPlot v.13 (Systat). The average inhibitory effect (IE) was calculated on the basis of individual PSP sweeps in the presence and absence of GABA_A_R-mediated signals (10–24 sweeps for Fig. 1; 2–10 sweeps for Fig. 8). The relationship between IE and EPSP amplitude was fit for individual cells with a single exponential function extrapolated between 1 and 25 mV EPSP amplitudes. The resulting fits are shown as mean ± s.e.m. (Fig. 3b, d, lower panel). Similarly, the relationship between PSP and EPSP amplitudes was fit for individual cells with a linear function, extrapolated and subsequently averaged (Supplementary Fig. 4). *E*_GABA_ was defined as the *x*-axis intercept of a third-order polynomial function fit to IPSP amplitude−voltage relationships (Fig. 2d, e). Analysis of KCC2 immunohistochemistry was performed using Fiji^76^. The perimeter of PV- and calbindin-expressing somata and dendrites was manually delineated and used to calculate the average fluorescence intensity of membrane-bound KCC2 labeling. For every optical section the background was subtracted and the results normalized to the total average KCC2 intensity.

Differences between two samples were assessed with a two-tailed unpaired or paired *t* test for independent and related samples, respectively. Nonparametric two-tailed Wilcoxon rank sum or signed-ranks tests were employed if normality tests failed. Multiple comparisons were performed using one- or two-way ANOVA on Ranks and post-hoc Dunn’s or Holm−Sidak method for pairwise comparisons unless otherwise stated. Significance levels are indicated as *p* values. Data are presented as mean ± s.e.m.

### Reporting summary

Further information on research design is available in the Nature Research Reporting Summary linked to this article.

## Supplementary information

Supplementary Information

Peer Review File

Reporting Summary

## Data Availability

The data that support the findings of this study are available from the corresponding authors upon reasonable request. Morphologies for cell models can be found in the ModelDB database (PVIs: https://senselab.med.yale.edu/ModelDB/ShowModel.cshtml?model=140789#tabs-1 and GCs: https://senselab.med.yale.edu/ModelDB/ShowModel.cshtml?model=95960). Codes for single-cell simulations will be stored in ModelDB: https://senselab.med.yale.edu/modeldb/.

## Code availability

All custom codes, protocols and mathematical algorithms used for data acquisition and analysis will be made available by the authors upon request.
