# Supplementary material for: Dendritic inhibition differentially regulates excitability of dentate gyrus parvalbumin-expressing interneurons and granule cells
Source: Nat Commun. 2019 Dec 5;10:5561. doi: 10.1038/s41467-019-13533-3 (PMC6895125; doi:10.1038/s41467-019-13533-3)
Supplement: Supplementary file 1 — Supplementary Information [file 41467_2019_13533_MOESM1_ESM.pdf]

Supplementary Material

Dendritic inhibition differentially regulates excitability of dentate gyrus parvalbumin-expressing interneurons and granule cells

Claudio Elgueta and Marlene Bartos

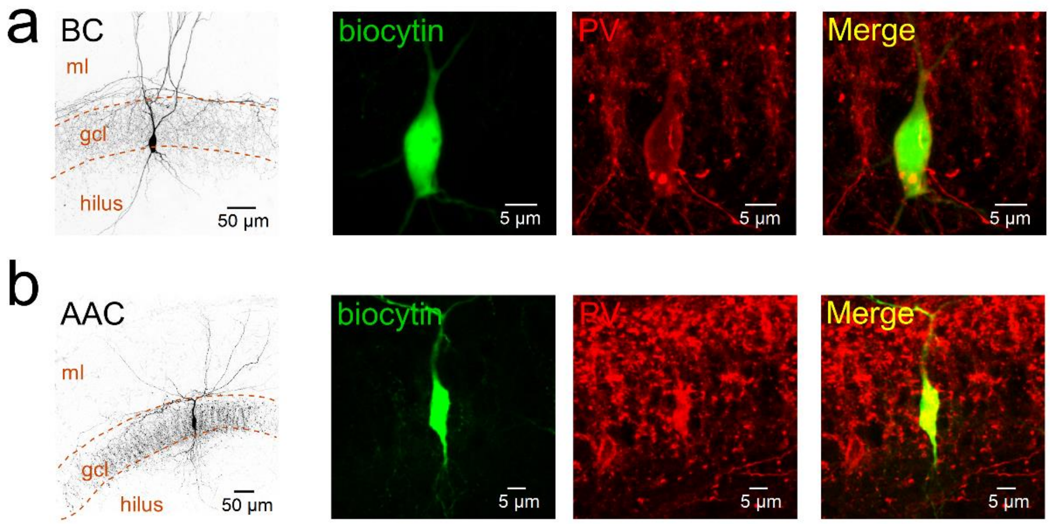

SUPPLEMENTARY FIGURE 1. Morphological and neurochemical identification of fast-spiking interneurons in the dentate gyrus as basket and axo-axonic cells. a, b Left, confocal image stacks of two intracellularly loaded cells with biocytin (see **Methods**) during whole-cell patch clamp recordings. Dendrites of these cells extend in the molecular layer (ml) and in the hilus while axons are distributed in the granule cell layer (gcl). Upper cell was identified as basket cell (BC) based on axon collaterals forming basket-like structures surrounding putative GC somata in the gcl, whereas the lower cell forms chandelier-like axonal processes, characteristic for axo-axonic cells (AACs). Right, visualization of antibody labeling against parvalbumin (PV) with Cy3-conjugated secondary antibody. Labelling reveals their PV-expressing nature (17 out of 19 tested cells were PV-positive).

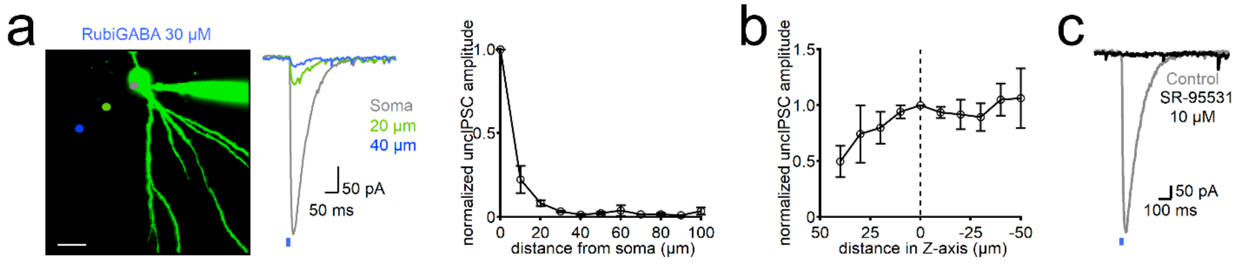

SUPPLEMENTARY FIGURE 2. Spatial resolution of 1-Photon RubiGABA uncaging. a Left, 2-Photon image stack of a GC during whole-cell recordings (recording pipette tip at the right) and schematic illustration of the location of three uncaging spots (gray, at the soma border; green 25 µm and blue 40 µm distance to the surface of the soma, scale bar 10µm). Middle, 3 representative $GABA_AR$-mediated currents (uncIPSCs) evoked by RubiGABA uncaging (30 µM, 0.5 ms, 0.5 mW laser power) at the 3 different distances from the GC soma shown on the left. Right, summary plot shows the normalized peak amplitude of $GABA_AR$-mediated currents evoked by RubiGABA uncaging as a function of distance from the soma (2 GCs, 1 PVI). **b** Summary plot showing that the response to RubiGABA uncaging remains constant when the focal plane is located deeper (negative values) than the dendritic position (0 µm) in the z-axis. **c** Representative average uncIPSCs before and after bath-application of SR-95531 10 µM (5 GCs, with CGP 2 µM in the control solution).

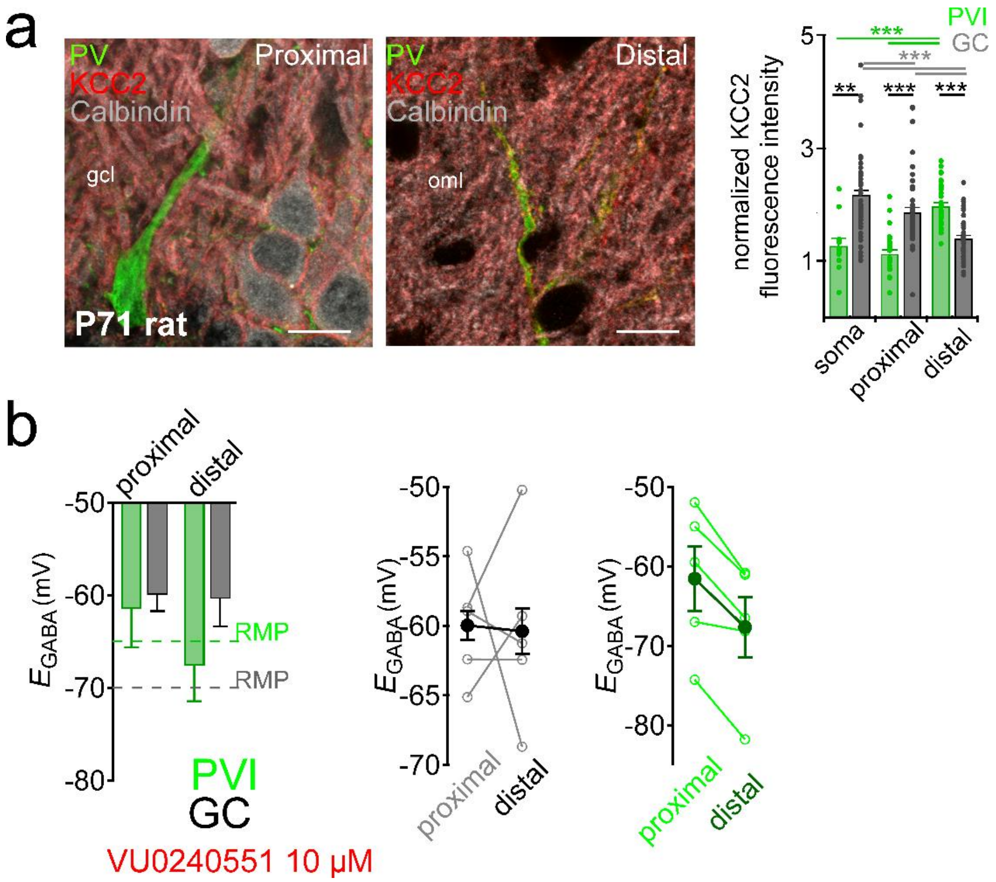

SUPPLEMENTARY FIGURE 3. KCC2 expression in PVI and GC dendrites and effects of KCC2 block on E_{GABA}. a Representative dentate gyrus slice from an adult (P71) rat brain immunolabelled against PV, KCC2 and calbindin (scale bar 10µm). Left image, granule cell layer (gcl); right image, outer molecular layer (oml). Bar graphs summarize the mean normalized KCC2 fluorescence intensity for PV and calbindin-expressing somata and dendrites (two-way ANOVA test with Holm-Sidak pairwise comparison). **b** Preincubation of slices with the KCC2 blocker VU0240551 (10 µM) induces a significantly more depolarized E_{GABA} in proximal and distal dendrites of GCs (5 GCs) and reduces the E_{GABA} difference between proximal and distal dendrites in PVIs (5 PVIs; compare to Fig. 2). Left, dashed lines represent the corresponding resting membrane potential (RMP; PVIs, -65 mV; GCs -70 mV). Right, individual open circles connected by lines represent individual experiments. Filled circles with lines and bars represent mean ± s.e.m. **, p ≤ 0.01; ***, p ≤ 0.001.

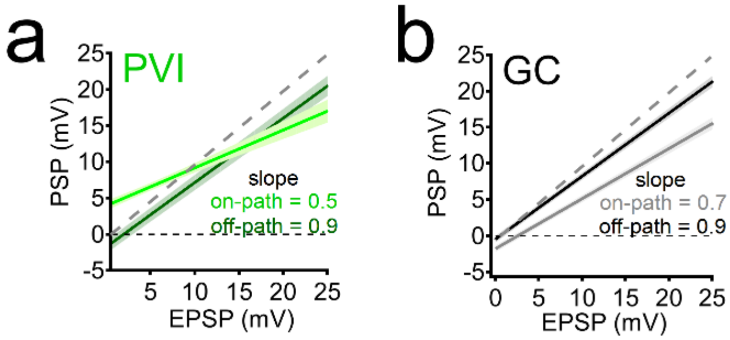

SUPPLEMENTARY FIGURE 4. Divisive and additive interactions of on- and off-path inhibition in PVIs and GCs. **a, b** Amplitude of postsynaptic potentials (PSPs) resulting from the interaction between inhibition and excitation were plotted as a function of the EPSP peak amplitude evoked by extracellular stimulation of the medial perforant path (somatodendritic distance ~150 µm). On- and off-path inhibition was evoked by RubiGABA uncaging of 7 randomly chosen spots targeting proximal dendrites on the level of the inner molecular layer (somato-dendritic distance of 25-75 µm) or distal dendrites on the level of the outer molecular layer (somato-dendritic distance > 200 µm) respectively. Data were fit to an extrapolated linear function for individual cells. Lines with shadows represent means ± s.e.m. for on- (bright green) and off-path (dark green) inhibition in PVIs, and on- (gray) as well as off-path (black) inhibition for GCs. Dashed line represent the identity line. Note, EPSPs with peak amplitudes < 9 mV were potentiated but reduced at > 9 mV by on-path inhibition in PVIs, resulting in a homogenization of PSP amplitudes. In contrast, EPSPs were always reduced in amplitude by on- and off-path inhibition in GCs.

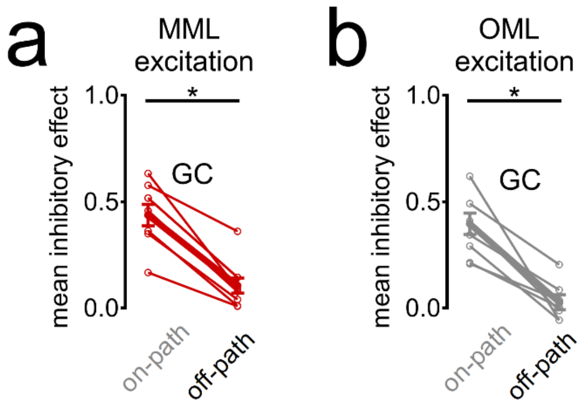

SUPPLEMENTARY FIGURE 5. Mild efficiency of off-path inhibition in controlling proximally and distally evoked excitatory signals at GC dendrites. **a, b** Extracellular stimulation pipette was placed either in the middle molecular layer (MML excitation; ~150 µm from the soma) or in the outer molecular layer (OML excitation; >200 µm from the soma) to evoke EPSPs in GCs. RubiGABA uncaging spots were placed either close to the soma (on-path, 25-75 µm) or at the distal tips of GC dendrites in the outer molecular layer (off-path, >200 µm). Note, on-path inhibition was always more efficient, independent of the precise EPSP induction site. Circles connected by lines represent individual experiments. Each circle is the mean of 20-50 traces. *, $p \leq 0.05$.

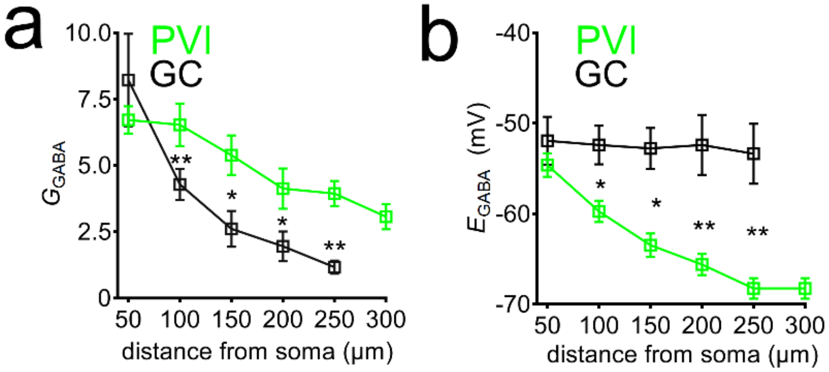

SUPPLEMENTARY FIGURE 6. Different distributions of $GABA_A$R-mediated conductances and E_{GABA} at PVI and GC apical dendrites. a, b Somatic whole-cell voltage-clamp recordings were performed from PVIs (green) and GCs (black) during RubiGABA uncaging at different positions along a single apical dendrite starting at 50 µm distance from the soma. The holding potential was systematically changed from -80 to -40 mV and the slope conductance (G_{GABA}) and the reversal potential of evoked $GABA_A$R-mediated signals (E_{GABA}) were calculated from the current-voltage relationship. The obtained G_{GABA} (**a**) and E_{GABA} (**b**) are plotted for PVIs (green) and GCs (black) as a function of somatic distance (6 PVIs and 5 GCs). Note, that during conditions of whole-cell recordings over similar periods of time (30.0 ± 1.13 and 28.0 ± 3.0 min for on- and off-path inhibition, respectively) using the same intracellular chloride-containing pipette solution, a gradient in E_{GABA} was maintained in PVIs. *, $p \leq 0.05$; **, $p \leq 0.01$.

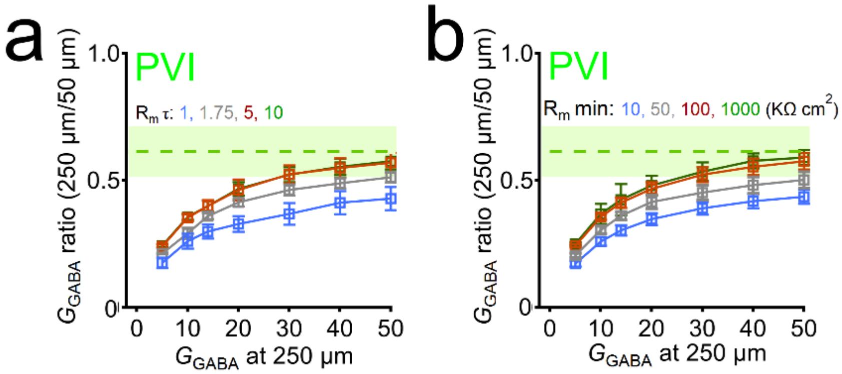

SUPPLEMENTARY FIGURE 7. Attenuation of distally evoked GABAergic signals in PVIs depends on the dendritic R_m gradient. a PVI single cell models were equipped with exponentially increasing membrane resistance (R_m) from 10 to 100 KΩ cm^2. This gradient was modified by systematically varying the constant τ from 1 to 10 (color code; 3 PVI models). Attenuation is depicted as the ratio of somatically measured G_{GABA} for signals induced at 250 and 50 µm somato-dendritic distance (14 nS at 50 µm). Note, that only under conditions of $\tau > 5$ and with distal G_{GABA} of > 40 nS we reproduced G_{GABA} ratios observed in vitro (green dashed line). **b** Same as (**a**) but with $\tau = 5$ and R_m at the dendritic tips was systematically increased to the color-coded values. Only under conditions of high distal G_{GABA} of > 40 nS and distal R_m > 100 KΩ, the measured G_{GABA} ratio reached values observed during in vitro experiments. Green dashed line with shaded area represent the experimentally defined mean ± s.e.m. G_{GABA} ratio. Squares with lines represent mean ± s.e.m.

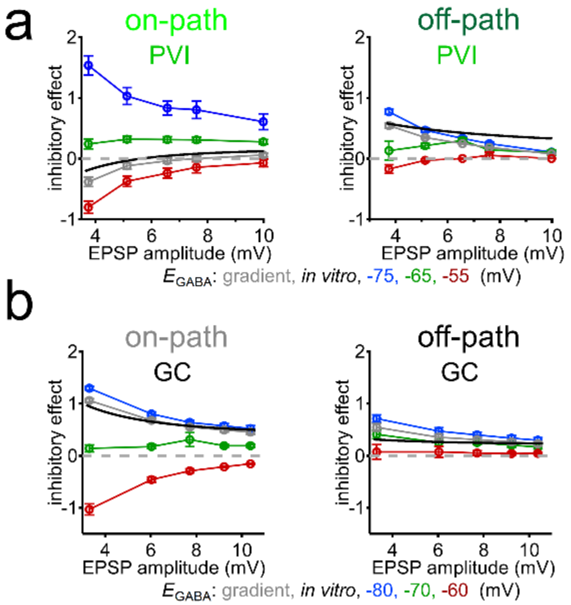

SUPPLEMENTARY FIGURE 8. The relation between inhibitory efficiency and the amplitude of excitatory signals is shaped by E_{GABA}. a In PVI models, an excitatory conductance (G_{exc}) was added at a somato-dendritic distance of 150 µm and step-wise increased to evoke EPSPs of varying amplitude. GABAergic inputs were activated either on- (~50 µm) or off-path (> 200 µm) relative to the EPSP induction site. E_{GABA} was changed from a linearly decreasing somato-dendritic gradient constrained by our in vitro data (gray traces) to uniform values of -75, -65 and -55 mV (blue, green and red, respectively; 3 PVI models). Black line depicts in vitro data (Fig. 3b, d). Note, the model with a linearly decreasing E_{GABA} could qualitatively reproduce our in vitro data for on- and off-path inhibition (black vs gray). **b** Same as in (**a**) for 3 GC models. Introducing our experimentally defined E_{GABA} gradient (Fig. 2e) or a constant E_{GABA} value of -80 mV qualitatively reproduced our in vitro data for on- and off-path inhibition (gray and blue vs black line). Squares with lines represent mean ± s.e.m.

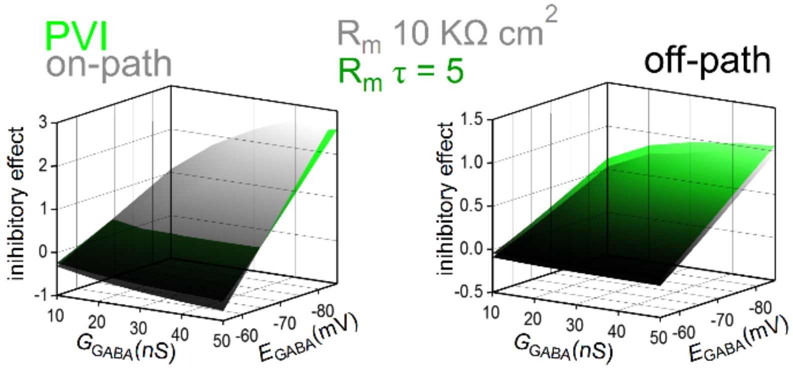

SUPPLEMENTARY FIGURE 9. The distribution of R_m along the somato-dendritic axis has a small influence on inhibitory efficiency in PVIs and GCs. Inhibitory effect (see Fig. 6e, legend for details) is plotted against G_{GABA} and E_{GABA} for on- (left, 50 µm from soma) and off-path inhibition (right, 250 µm from soma) in PVIs. The exponentially increasing R_m from the soma to distal dendrites used as basic condition in our single cell simulations (green surfaces; R_m = 10 - 100 KΩ cm^2, τ = 5, 3 model PVIs) was changed to a model with constant R_m value (gray surfaces; R_m = 10 KΩ cm^2). Inhibitory effects were measured in relation to EPSPs evoked at somato-dendritic distances of 150 µm (excitatory conductance 5 ns; EPSP amplitude of 5.1 mV, measured at the soma). Note, R_m had a negligible influence on inhibitory effects.

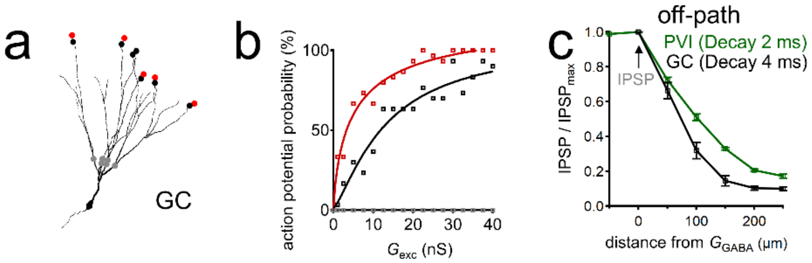

SUPPLEMENTARY FIGURE 10. Proximal inhibition is more efficient than distal inhibition in controlling action potential generation evoked by distal inputs in GCs and attenuation of inhibitory signals in GCs and PVIs. a Morphology of a representative GC used for single cell simulations. Five excitatory inputs where located at the tips of apical dendrites of GC models (red filled circles). G_{GABA} was applied at seven distal distributed either close to the soma (gray filled circles) or at the distal regions of dendrites (black filled circles). **b** Action potentials were generated by systematically rising the magnitude of excitatory conductances (G_{exc}), which induced a continuous increase in action potential probability (red squares). When GABAergic inputs close to the soma were activated, action potential generation was completely abolished (gray squares). In contrast, when distal G_{GABA} were introduced near G_{exc} locations (5 µm distance) only a mild effect on the generation of action potentials was observed (black squares). **c** Attenuation of distally evoked IPSPs was evaluated in GCs and PVIs (3 and 5 model cell, respectively) using fast unitary decay time constants of the $GABA_A$ receptor-mediated conductance (GC: 4 ms, PVI: 2 ms[43]). Squares with lines represents mean ± s.e.m. Lines in (**b**) represent sigmoid fits to the mean action potential probability.

Supplementary Table 1. Distribution of conductances in PVI model cells.

$\bar{g}$ (mS cm^{-2})	axon	soma	dendrites
Na	25 (85)	25 (55)	1 (0)
K	50	20	20
I_h	0.001	0.001	0.001

Distributions were based on Nörenberg et al. (2010)[22], Hu et al. (2010)[24] and Elgueta et al. (2015)[55]. Values in brackets depict conductance densities ($\bar{g}$) used for simulations shown in Fig. 7.

Supplementary Table 2. Distribution of conductances at the different compartments of GC model cells.

$\bar{g}$ (mS cm^{-2})	axon	soma	gcl	proximal dendrite	medial dendrite	distal dendrite
Na	315	180	19.5	19.5	12	
fK_{DR}	22.4	13	3.2	3.2	0.8	0.8
sK_{DR}	-	2.4	2.4	2.4	2.4	0.32
K_A	4	12	-	-	-	-
CaT	-	0.1	0.5	0.5	1	1
CaN	-	2	1	1	1	1
CaL	-	1	15	15	1	1
B_K	-	0.09	0.15	0.15	0.036	0.036
S_K	-	0.1	0.01	0.01	-	-
Un	-	200	-	-	-	-
sAHP	-	10	-	-	-	-

Distributions of conductance density ($\bar{g}$) are based on Mateos-Aparicio et al. (2014)[54].
