# Supplementary material for: Dendritic inhibition differentially regulates excitability of dentate gyrus parvalbumin-expressing interneurons and granule cells
Source: Nat Commun. 2019 Dec 5;10:5561. doi: 10.1038/s41467-019-13533-3 (PMC6895125; doi:10.1038/s41467-019-13533-3)
Supplement: Supplementary file 3 — Reporting Summary [file 41467_2019_13533_MOESM3_ESM.pdf]

nature research

Corresponding author(s): Marlene Bartos

Last updated by author(s): Oct 24, 2019

Reporting Summary

Nature Research wishes to improve the reproducibility of the work that we publish. This form provides structure for consistency and transparency in reporting. For further information on Nature Research policies, see Authors & Referees and the Editorial Policy Checklist .

Statistics

For all statistical analyses, confirm that the following items are present in the figure legend, table legend, main text, or Methods section.

n/a	Confirmed	
☐	☒	The exact sample size (*n*) for each experimental group/condition, given as a discrete number and unit of measurement
☐	☒	A statement on whether measurements were taken from distinct samples or whether the same sample was measured repeatedly
☐	☒	The statistical test(s) used AND whether they are one- or two-sided<br>*Only common tests should be described solely by name; describe more complex techniques in the Methods section.*
☒	☐	A description of all covariates tested
☐	☒	A description of any assumptions or corrections, such as tests of normality and adjustment for multiple comparisons
☐	☒	A full description of the statistical parameters including central tendency (e.g. means) or other basic estimates (e.g. regression coefficient) AND variation (e.g. standard deviation) or associated estimates of uncertainty (e.g. confidence intervals)
☒	☐	For null hypothesis testing, the test statistic (e.g. *F*, *t*, *r*) with confidence intervals, effect sizes, degrees of freedom and *P* value noted<br>*Give P values as exact values whenever suitable.*
☒	☐	For Bayesian analysis, information on the choice of priors and Markov chain Monte Carlo settings
☒	☐	For hierarchical and complex designs, identification of the appropriate level for tests and full reporting of outcomes
☒	☐	Estimates of effect sizes (e.g. Cohen's *d*, Pearson's *r*), indicating how they were calculated

Our web collection on statistics for biologists contains articles on many of the points above.

Software and code

Policy information about availability of computer code

Data collection	Fpulse 2.37 (Igor-custom based script from Cambridge electronic design acquisition boards, created by Mr. Ulrich Fröbe, Freiburg University), Igor Pro6.0 (Wavemetrics), Heka Pulse (HEKA), MES 4.0 (Femtonics), Neuron (yale)
Data analysis	Igor Pro 7.0 (Wavemetrics), Ola analysis 3.15 (custom igor-based analysis routines, created by Dr. Claudio Elgueta, Freiburg University), Matlab 2017 (Mathworks), Fiji-Imagej, Sigma-Plot (Systat)

For manuscripts utilizing custom algorithms or software that are central to the research but not yet described in published literature, software must be made available to editors/reviewers. We strongly encourage code deposition in a community repository (e.g. GitHub). See the Nature Research guidelines for submitting code & software for further information.

Data

Policy information about availability of data

All manuscripts must include a data availability statement. This statement should provide the following information, where applicable:

- Accession codes, unique identifiers, or web links for publicly available datasets
- A list of figures that have associated raw data
- A description of any restrictions on data availability

All electrophysiological, imaging and computational data are available upon request.

Field-specific reporting

Please select the one below that is the best fit for your research. If you are not sure, read the appropriate sections before making your selection.

☒ Life sciences ☐ Behavioural & social sciences ☐ Ecological, evolutionary & environmental sciences

Life sciences study design

All studies must disclose on these points even when the disclosure is negative.

Sample size	No sample size calculation was performed. The number of cells experimentally tested to address a given question was equal or larger than 6 and correlated with the expected effect size. In case of single cell computations, the number of single cell simulations dependent on the number of reconstructed parvalbumin-expressing interneurons (PVIs) and granule cells (GCs) of the dentate gyrus and was n = 5 for PVIs and 5 for GCs.
Data exclusions	Cells were excluded if their identity was not confirmed physiologically and morphologically, and if their resting membrane potential was too depolarized (more than -50 mV for PVIs, more than -70 mV for GCs), which indicates that cells are in unphysiological conditions.
Replication	In vitro experiments were always repeated in slice preparations from different animals.
Randomization	Randomization was not applicable because two conditions have been compared.
Blinding	Blinding was not applicable because two recognizable cell types have been compared.

Reporting for specific materials, systems and methods

We require information from authors about some types of materials, experimental systems and methods used in many studies. Here, indicate whether each material, system or method listed is relevant to your study. If you are not sure if a list item applies to your research, read the appropriate section before selecting a response.

Materials & experimental systems

n/a	Involved in the study	
☐	☒	Antibodies
☒	☐	Eukaryotic cell lines
☒	☐	Palaeontology
☐	☒	Animals and other organisms
☒	☐	Human research participants
☒	☐	Clinical data

Methods

n/a	Involved in the study	
☒	☐	ChIP-seq
☒	☐	Flow cytometry
☒	☐	MRI-based neuroimaging

Antibodies

Antibodies used	Parvalbumin PV27 (Swant), Donkey Cy3-conjugated anti-rabbit (Jackson Immunoresearch); calbindin guinea pig (Synaptic Systems); KCC2 rabbit (Milipore); Alexa Fluor-647 anti-guniea pig (Jackson ImmunoResearch) host donkey.
Validation	Parvalbumin antibody PV27 and 235 were validated by using Parvalbumin knockout animals (https://www.swant.com/pdfs/PV27_Rabbit_anti_Parvalbumin.pdf and https://www.swant.com/pdfs/Monoclonal_parvalbumin_235.pdf). Cy3-conjugated anti-rabbit (Jackson Immunoresearch) was validated by ELISA (https://www.dianova.com/downloads/Jackson/711-165-152.pdf). Calbindin guinea pig was purified with the immunogene (https://www.sysy.com/products/calbindin/facts-214005.php). KCC2 was routinely evaluated by western blot in rat brain membrane preparations (http://www.merckmillipore.com/DE/de/product/Anti-K+-Cl-Cotransporter-KCC2-Antibody,MM_NF-07-432). Alexa Fluor-647 anti-guinea pig Alexa Fluor-488 anti mouse were tested using immunoelectrophoresis and/or ELISA to test their reaction with whole molecule guinea pig IgG or whole molecule mouse IgG respectively (https://www.jacksonimmuno.com/catalog/products/106-605-003#, https://www.jacksonimmuno.com/catalog/products/115-545-003#)

Animals and other organisms

Policy information about studies involving animals; ARRIVE guidelines recommended for reporting animal research

Laboratory animals	Wistar rats were obtained from Charles River. Somatostatin-CRE mice were obtained from JAX mice (STOCK: 013044).
Wild animals	Not applicable.
Field-collected samples	Not applicable.
Ethics oversight	Regional council ('Regierungspräsidium') Freiburg (Germany). Licenses number: X-16/306S, X-13/03S and G-15/106.

Note that full information on the approval of the study protocol must also be provided in the manuscript.
