# Supplementary material for: Dendritic inhibition differentially regulates excitability of dentate gyrus parvalbumin-expressing interneurons and granule cells
Source: Nat Commun. 2019 Dec 5;10:5561. doi: 10.1038/s41467-019-13533-3 (PMC6895125; doi:10.1038/s41467-019-13533-3)
Supplement: Supplementary file 2 — Peer Review File [file 41467_2019_13533_MOESM2_ESM.pdf]

Reviewers' comments:

Reviewer #1 (Remarks to the Author):

In this paper, the authors describe the interaction between excitation and on-path (or perisomatic) or off-path (or dendritic) inhibition in granule cells and PV-positive interneurons in the dentate gyrus of young, 3-4 week-old, rats. Technique-wise, they use an impressive variety of methods. They combine whole-cell recordings with glutamate microiontophoresis, RubiGABA uncaging and also some optogenetics and also perform computational modeling. They find that, in granule cells, only on path inhibition can suppress action potential firing by hyperpolarizing the neuron back to the GABA reversal potential. In PV-positive interneurons, both on-path and off-path inhibition can prevent action potential firing, albeit through different mechanisms of either shunting (on-path) and hyperpolarization (off-path). The experimental results are recapitulated in single cell computer models when implementing different G(GABA) and E(GABA) gradients in the granule cells and PV interneurons. I find this study very interesting; however, I have several questions or comments that the authors, in my opinion, should address:

1) I did not find in the paper any description of how the authors determined the amount of glutamate applied, the laser power used for the GABA uncaging or the amount of extracellular stimulation for the gramicidin experiment. How do these levels of excitation and inhibition compare to physiologically observed ones? The authors should clarify how they decided on these experimental parameters.

2) Along the same line, it would be great to have an idea whether the amount of uncaged RubiGABA and the distance from the uncaging location to the dendrite (or to GABAergic synapses for that matter) are comparable in proximal vs. distal dendrites? All of the computational modeling results rely on this being the case.

3) It is very interesting that there is this 10 mV difference in the GABA receptor reversal potential between the proximal and distal part of the dendrite of the PV interneurons and that there is, as a consequence, this differential effect of GABA on the membrane potential. This finding suggests that the chloride concentration gradient between extra- and intracellular space is reversed in the proximal part compared to the distal one. It would be useful to validate this result by (1) applying bicuculline to show that the RubiGABA evoked PSPs are indeed GABA(A)-receptor dependent and (2) by pharmacologically changing the chloride gradient (e.g., block of K-Cl-cotransporters) also in the distal dendrites and checking that this change is detectable in the whole-cell recordings. Is there any evidence (e.g., using immunohistochemistry) that there is a soma-dendrite gradient of K-Cl-cotransporters or other chloride co-transporters specifically in the PV interneurons in dentate gyrus?

4) One of the predictions of this work would be, I think, that SOM interneuron activation leads to reduced firing rate in PV interneurons, which reduces proximal inhibition in granule cells and therefore increases action potential firing in granule cells. Thus, in the SOM stimulation experiment shown in Fig 7f, I would have expected a maybe small, but detectable increase in the granule cell firing rate. However, if anything, there is a slight decrease in granule cell firing. Why this discrepancy? Is there potentially a rat-mouse species difference (slice experiments were done in rat slices, SOM optogenetic stimulation was done in SOM-cre mice)?

Minor points:

1) What is the function of the off-path inhibition in granule cells? The authors mention, sort of in passing in the discussion, that they might influence local dendritic excitation and plasticity.
2) I assume that all of the traces shown in the paper are from individual trials, e.g., fig. 1 c and d. Is there any systematic variability between responses to single uncaging pulses (with a 2 ms inter-pulse interval), for example over time? Do the authors apply several pulses at the same dendritic location?
3) Fig. 7f, the figure legend does not seem to fit the panel.

4) There are several typos, for example in the first paragraph of the introduction.

Reviewer #2 (Remarks to the Author):

The present MS described in vitro electrophysiology experiments addressing the effectiveness of dendritic inhibition in two distinct cell types (parvalbumin expressing INs: PVIs and granule cells: GCs) of the dentate gyrus. The authors performed extracellular stimulation, GABA uncaging and somatic recordings to demonstrate that distal, off-path inhibition is very powerful in PVIs, but not in GCs. With combined experimental and modelling approaches they demonstrate that this is mainly the consequence of different EGABA and GGABA in these cell types. Most of the results are convincing, revealing rather unexpected roles of distal inhibition and the cell type-dependent differences in its effectiveness. There are a number of issues the authors should address prior to publications.
The authors elegantly demonstrate that the EGABA is not uniform in PVIs; having a significantly more depolarized value in proximal, compared to distal locations. These results were obtained correctly with Gramicidin perforated patch experiments. However, in some experiments the authors claimed to use whole-cell recordings and applied an intracellular [Cl-] that resulted in a EGABA of ~63 mV. ('EGABA was kept close to values 111 previously described for dentate gyrus PVIs and GCs, by loading cells during recordings with 112 a pipette solution that resulted in a chloride reversal potential of -63.3 mV.') How did the authors decide what EGABA (proximal or distal) should be 'reproduced' in the whole-cell experiments? What was the intracellular [Cl-] in experiments described in Fig 3-8?
The authors used realistic cell modelling to predict the dendritic distribution of GGABA. Here, a key parameter, namely the Ra in the different cell types, is unknown. In addition, it is known that the deactivation time constant of the GABAA receptors are very different in these two cell types (much faster in PVIs), suggesting a more pronounced attenuation. Have the authors considered the effects of different inhibitory conductance kinetics?
Along the same line, the reviewer would have liked to see independent evidence for the increased GGABA in the distal dendrites, such as synapse density and/or synapse size differences.
The plots in figure 6e is hard to read and interpret. The authors should consider changing it to a more conventional 3D plot.
The experiments described in figure 7f is very informative, but the number of repetition is very low. The reviewer suggests to increase the 'N' of these experiments preferably in a way that GCs and PVIs are recorded simultaneously, thus controlling for the effectiveness of the light-evoke GABA release.

Reviewer #3 (Remarks to the Author):

The study by Elgueta and Bartos presents very interesting experimental and computational data on the efficacy of dendritic GABAergic inhibition in dentate PVIs and GCs. The data quality is excellent, and the conclusions are well supported. Overall, they report distinct gradients of inhibitory conductance along the dendritic lengths of these two cell classes, along with distinct reversal potential. These differences lead to remarkable differences in how the spatial alignment of inhibition and excitation shape synaptic integration. The findings are further supported by computational modeling. Overall, I am quite enthusiastic about this work. However, there are a few points that I think should be addressed to increase the impact of the study.

1. The biggest criticism is of course, that most data are derived from non-physiological synaptic stimulation. Neither local puffing nor 1p uncaging closely mimics the spatiotemporal spread of synaptically released transmitter and activation of postsynaptic receptors. Indeed, the potential involvement of extrasynaptic receptors in the present study (especially for GABARs) raises the question of whether the results would hold true if only synaptic inhibitory receptors were activated. The optogenetic experiments with SOM-INs mitigate this to some extent. However, this issue is not raised at all in the manuscript. At a minimum, a thorough discussion of this concern should be added.

However, it would be much preferable to add more precise stimulation data – e.g., 2p GABA uncaging (though the reviewer acknowledges the technical challenges of this approach).

2. The experiments are done in relatively young animals (<P30). There is substantial maturation of GABAergic synapses over the 3-5 weeks of life. It would be interesting to add some basic experimental characterization of adult animals (>P60) to confirm if the basic findings hold true.

3. The conclusions rest entirely on physiological and computational data. While these results are compelling, they don't distinguish a number of possibilities (differences in receptor subunit composition or synaptic density along the somatodendritic axis). The addition of anatomical data (e.g., immunolabeling gephyrin or better, viral expression of tagged-gepyhrin) to characterize the distribution of putative inhibitory synapses in both cell types would be of enormous benefit.

4. While some studies are cited, the introduction should really expand a bit on the now substantial literature on dendritic inhibition to set up the overall study. Work from the Higley, Carter, Bonhoeffer, Larkum, and Segev labs have all considered dendritic inhibition extensively.

We thank the senior editor and the referees for their constructive and insightful comments on our manuscript. We followed the proposals made by the referees and responded point-by-point to their comments.

Reviewer #1:

We thank Reviewer #1 for praising the '*impressive'* variety of the methods applied in this '*very interesting'* study. However, the reviewer had several questions and comments, which we address in the following:

Main Comments:

1) I did not find in the paper any description of how the authors determined the amount of glutamate applied, the laser power used for the GABA uncaging or the amount of extracellular stimulation for the gramicidin experiment.

The Methods section on page 11, chapter '*Glutamate microiontophoresis and GABA uncaging',* states details on the concentration of glutamate and RubiGABA as well as the laser power: '…*For GABA uncaging, slices were perfused with an extracellular solution containing RubiGABA (30 µM, prepared from a 1 mM stock dissolved in HEPES-buffered and Na^+-rich solution and kept it at -80°C) with a total volume of 8-15 ml (~32°C)….*'. Following the reviewer's suggestion we improved the methods section by stating in the revised manuscript on page 11, 1st and page 12, 1st paragraph, that in case of RubiGABA uncaging, we maintained stimulation intensity constant across dendritic compartments and cell types, by keeping the duration (0.5 ms) and the power of the laser (0.5 mW, measured at the exit of objective lens), and the concentration of RubiGABA in the external solution, constant. This experimental setting resulted in robust responses in GCs and PVIs without inducing photo-toxicity after repeated stimulations. Photo-toxicity would have been observed as a change in dendritic morphology such as the formation of dendritic varicosities. To measure the reversal potential of synaptically evoked IPSPs during perforated patch (gramicidin) whole-cell recordings, extracellular stimulation of GABAergic inputs was performed at constant stimulation intensity (50 V) and duration (0.1 ms). The resistance of glass pipettes, which were used for extracellular stimulation, after loading with a Na^+-enriched HEPES-buffered solution, was ~1 MΩ.

How do these levels of excitation and inhibition compare to physiologically observed ones? The authors should clarify how they decided on these experimental parameters.

Application of glutamate by microiontophoresis at a somatic distance of ~150 µm resulted in EPSPs with an amplitude of 4.0 ± 0.7 mV recorded at PVI and 7.3 ± 0.6 mV recorded at GC somata. Perforant path-mediated unitary EPSPs have so far not been experimentally measured in PVIs and they have been estimated in GCs based on glutamate uncaging at individual spines at the level of the middle molecular layer (~150 µm somatic distance) to ~1 nS, producing a 0.6 - 1.0 mV depolarization at the GC soma (Schmidt-Hieber et al. J Neurosci 27, 2007; Krueppel et al., Neuron 71, 2011). Based on this published data, our experiments shown in **Fig. 1** reproduce the simultaneous stimulation of ~7 unitary synaptic inputs in GCs. If we expect the same local excitatory conductance in PVIs (Ewell & Jones, J Neurosci 30, 2010), microiontophoretic stimulation will mimic the activation of ~4 synaptic inputs in PVIs. This information is added to the results section on page 3, 2nd paragraph.

Paired recordings revealed that the $GABA_A$ receptor-mediated unitary conductance evoked by a perisomatic synapse is 6.8 nS for PVI-GC (Kraushaar & Jonas, J Neurosci 20, 2000), and 7.6 nS for PVI-PVI synapses (Bartos et al., J Neurosci 21, 2001). Using our detailed, morphology-based single cell GC and PVI models, we calculated that activation of a 10 nS and 14 nS $GABA_A$ receptor-mediated conductance at a distance of 50 µm to the soma of PVIs and GCs, respectively, reproduced average uncIPSP peak amplitudes experimentally measured in GCs and PVIs upon RubiGABA uncaging at the same somatic distance of 50 µm (shown in **Fig. 5)**. Thus, these values correspond to the activation of two presynaptic GABAergic interneurons converging onto one GC and one PVI. This information is added to the Methods section of the revised manuscript on page 14, 1st paragraph.

The decision of choosing these parameters was the best compromise between physiologically plausible evoked PSPs, while obtaining reliable, clearly visible and precisely localized activation of excitatory and inhibitory inputs. Moreover, for GABAergic signals, we wished to have comparable numbers of converging inputs across cell types and cellular compartments.

2) Along the same line, it would be great to have an idea whether the amount of uncaged RubiGABA and the distance from the uncaging location to the dendrite (or to GABAergic synapses for that matter) are comparable in proximal vs. distal dendrites? All of the computational modeling results rely on this being the case.

We used short stimulation duration and mild laser power for RubiGABA uncaging. Under these conditions, we observed no signs of photo-damage. We therefore stimulated in direct proximity to the dendrites at both distal and proximal locations of PVIs and GCs. Moreover, for each cell, we stimulated dendrites located at similar depths in the slice (maximum z-axis distance between stimulated dendrites 35 µm). As shown now in **Fig. S2b**, in this range of tissue depth IPSPs with comparable amplitude were evoked, indicating similar amounts of uncaged RubiGABA. This is now specified in the methods section on page 12, 1st paragraph. Thus, we strongly expect that the amount of uncaged GABA was similar for the different uncaging positions and cells.

3) It is very interesting that there is this 10 mV difference in the GABA receptor reversal potential between the proximal and distal part of the dendrite of the PV interneurons and that there is, as a consequence, this differential effect of GABA on the membrane potential. This finding suggests that the chloride concentration gradient between extra- and intracellular space is reversed in the proximal part compared to the distal one. It would be useful to validate this result by (1) applying bicuculline to show that the RubiGABA evoked PSPs are indeed GABA(A)-receptor dependent and (2) by pharmacologically changing the chloride gradient (e.g., block of K-Cl-cotransporters) also in the distal dendrites and checking that this change is detectable in the whole-cell recordings. Is there any evidence (e.g., using immunohistochemistry) that there is a soma-dendrite gradient of K-Cl-cotransporters or other chloride co-transporters specifically in the PV interneurons in dentate gyrus?

We thank the reviewer for these interesting experimental proposals. (***a***) We applied, as suggested by the reviewer, the $GABA_A$-receptor antagonist SR-95531 and show that it effectively blocked IPSCs evoked by RubiGABA uncage (5 GCs). This data are added to the revised **Fig. S2c** and in the main text on page 3, 2nd paragraph.

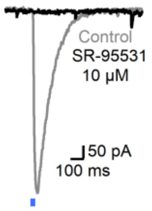

Supplementary Figure 2c: Gabazine (SR-95531) blocked uncIPSCs in GCs. Grey traces, averages of individual uncIPSCs; black trace after SR 95531 bath application (10 µM).

(***b***) Following the reviewers suggestion we tested the involvement of K-Cl cotransporter KCC2 using perforated-patch whole-cell recordings during bath application of the specific KCC2 blocker VU0240551 (10 µM). Pre-incubation of slices with this drug had a significant

effect on E_{GABA} of both proximal and distal GABAergic signals in GCs. E_{GABA} was significantly more depolarized during KCC2 block compared to control conditions (for proximal IPSPs during KCC2 block: -60.0 ± 1.8 mV vs control IPSCs -78.2 ± 1.0 mV; for distal IPSPs during KCC2 block: -60.4 ± 3.0 mV vs control IPSPs -74.4 ± 1.6 mV; 5 and 6 GCs, respectively; p = 0.0005 and 0.008, respectively). This effect can be explained by intracellular chloride accumulation in proximal and distal dendritic compartments in presence of the KCC2 blocker. In the case of PVIs, block of KCC2 resulted in a loss of the significant difference in E_{GABA} between proximal and distal sites (p = 0.3; see below new **Fig. S3b**). However, the effect of the blocker was smaller than in GCs. This might be explained by the expression of potentially different KCC2 isoforms (Uvarov et al., J Biol Chem 282, 2007; Chamma et al., Frontier in Cellular Neurosci, 2012), by different affinities for the applied antagonist between the two cell types or by additional chloride extrusion mechanisms. The newly obtained data are added to the main text on page 4, 3rd paragraph and are shown in **Fig. S3b** of the revised manuscript.

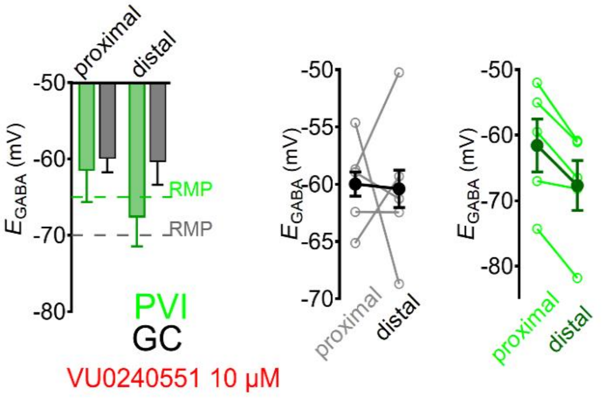

Supplementary Figure 3b: Preincubation of hippocampal slice preparations with the KCC2 blocker VU0240551 (10 μM) induces a significantly more depolarized E_{GABA} in proximal and distal dendrites of GCs (5 GCs) and reduces the E_{GABA} difference between proximal and distal dendrites in PVIs (5 PVIs). *Left*, Striped lines represent the corresponding resting membrane potential (RMP; PVIs, -65 mV; GCs -70 mV). *Right*, individual open circles connected by lines represent individual experiments. Filled circles with lines represent mean ± SEM.

(***c***) We performed antibody labelling against parvalbumin (PV), KCC2 and calbindin as a marker for mature GCs in the ventral dentate gyrus of P24-old rats. We found that KCC2 antibody-labelling was constrained to the membrane of calbindin-expressing mature GCs located in the outer half but not in the inner half of the granule cell layer, close to the hilus, which contains putative adult-born immature GCs. Interestingly, the membrane of somata and proximal dendrites of PVIs were not surrounded by KCC2 labelling. In contrast, distal PV-expressing dendrites showed strong KCC2 immuno-labelling. These data suggest a differential subcellular expression of K-Cl cotransporters along the somato-dendritic axis of PVIs and are in line with our results showing different E_{GABA} values between proximal and distal PVI dendrites and are now included in the main text on page 4, 3rd paragraph and **Fig. 2g-i.** These data were reproduced for adult (P71) rats and shown in **Fig. S3a**.

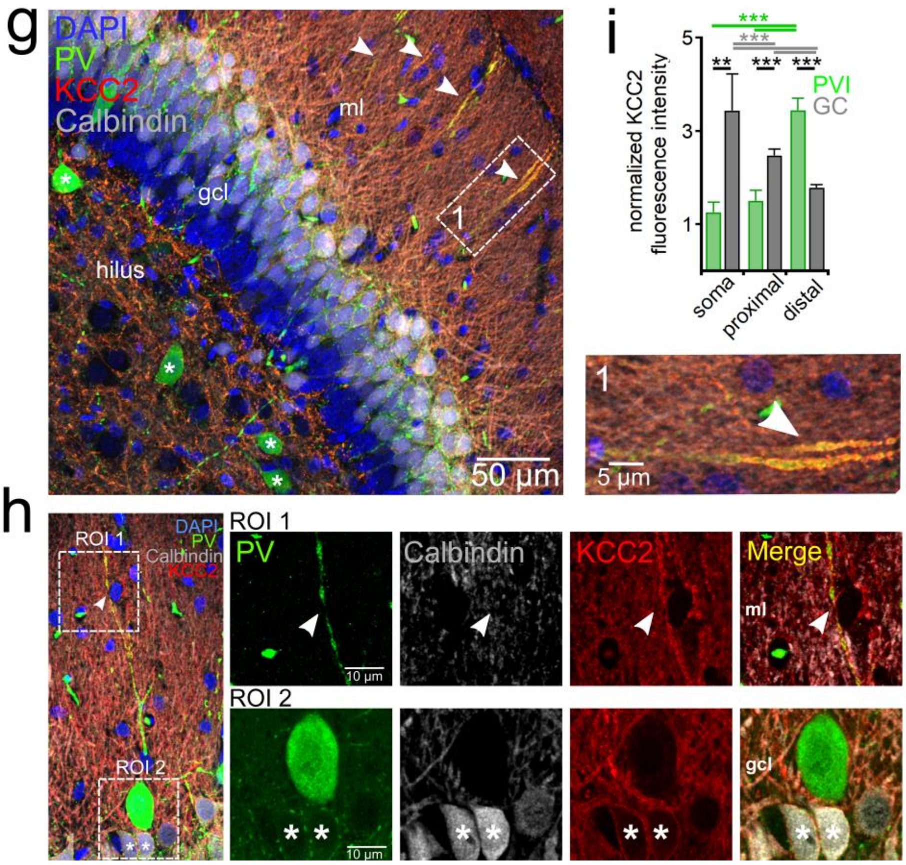

Figure 2g-h: (***g***) Maximum intensity projection from 5 x 0.67 µm optical sections of a ventral dentate gyrus slice from an adolescent (P24) rat stained for parvalbumin (PV), KCC2 and calbindin, and counterstained with DAPI. *Note*, that in the granule cell layer (gcl) membrane-bound KCC2 is expressed at the soma of calbindin-positive GCs but not at immature calbindin-negative GCs. In contrast, somata of PV-expressing interneurons rarely show membrane-bound KCC2 (asterisks), while PV-labelled dendrites in the molecular layer (ml) demonstrate strong expression of the transporter (arrowheads). *Inset lower right*, magnified section depicted by a white square (1). (***h***) Maximum intensity projection of 11 x 0.36 µm optical sections from one PVI. ROI-1 and -2 are shown at higher magnification in the right panels. *Note*, KCC2 is expressed at the border of calbindin-expressing GC somata (asterisks) but not at PVI cell bodies (ROI 2), while distal dendrites of the same PVI show strong KCC2 staining (ROI 1, arrowhead). (***i***) Bar graphs summarize normalized fluorescence intensity of KCC2 antibody labelling at the soma, proximal and distal dendrites of PVIs and calbindin-expressing GCs. *, $p \leq 0.05$; **, $p \leq 0.01$; ***, $p \leq 0.001$.

4) One of the predictions of this work would be, I think, that SOM interneuron activation leads to reduced firing rate in PV interneurons, which reduces proximal inhibition in granule cells and therefore increases action potential firing in granule cells. Thus, in the SOM stimulation experiment shown in Fig 7f, I would have expected a maybe small, but detectable increase in the granule cell firing rate. However, if anything, there is a slight decrease in granule cell firing. Why this discrepancy? Is there potentially a rat-mouse species difference (slice experiments were done in rat slices, SOM optogenetic stimulation was done in SOM-cre mice)?

We thank the reviewer for emphasizing this point. The scenario proposed by the reviewer requires a given level of activity of PVIs and GCs in slice preparations of the dentate gyrus driven by spontaneous excitatory perforant path-mediated inputs and strong connectivity from PVIs onto GCs. However, GC activity under *in vitro* conditions is low and connectivity is reduced compared to *in vivo* conditions due to tissue slicing. Although some PVIs are active in the slice preparation as indicated by the recorded spontaneous IPSCs in GCs with

fast kinetic properties, most of the PVIs and GCs show sparse spontaneous action potential generation *in vitro*. We therefore believe that under our experimental conditions we mainly observed the direct effect of dendritic inhibition onto GC activity and not the entire microcircuit-mediated effect, namely the inhibitory influence of dendritic inhibition onto PVI discharges and in turn the influence of reduced PVI discharges and reduced GABAergic transmission onto GC activity.

Although we cannot fully exclude species-specificities, previous work in the mouse and rat dentate gyrus, including our own studies (functional and dynamic properties of synapses targeting GCs and PVIs, their intrinsic characteristics and connectivity; Bartos et al., J Neurosci 2001, PNAS USA 2002), points to similar functional characteristics of the dentate gyrus microcircuit in mice and rats. Moreover, and as pointed out by the reviewer, we performed only the optogenetic experiments in mice.

Minor comments:

1) What is the function of the off-path inhibition in granule cells? The authors mention, sort of in passing in the discussion, that they might influence local dendritic excitation and plasticity.

Previous anatomical studies by Buckmaster et al. (J Comp Neurol 445, 2002) showed that 55% of GABAergic synapses in the outer molecular layer were located at GC spines. We believe that the combination of a shunting E_{GABA}, the passive properties of GCs and the localization of distal inhibitory inputs on spines lead to a compartmentalized function of dendritic inhibition in GCs. Spatially-localized inhibition might regulate calcium signaling and synaptic plasticity, as it has been suggested for principal cells in the prefrontal cortex (Chiu et al., Science, 340, 2013). We have addressed this issue in the discussion on page 10, 2nd paragraph.

2) I assume that all of the traces shown in the paper are from individual trials, e.g., fig. 1 c and d. Is there any systematic variability between responses to single uncaging pulses (with a 2 ms inter-pulse interval), for example over time? Do the authors apply several pulses at the same dendritic location?

Yes, in the entire manuscript we show individual traces. Variability of uncaging responses was low within cells (see below, the randomly chosen example in the **Figure for reviewer 1**). Multiple trials were applied to the same dendritic position in **Fig. 1 & 5.** However, the minimum inter-pulse uncage interval in those experiments was 12 s for a particular dendritic position. Under these conditions, we did not observe any decline in the amplitude of evoked IPSPs. The 2 ms inter-pulse interval to which the reviewer is referring to, was only used during sequential stimulation of seven different dendritic segments in one cell (**Fig. 3, 4, 8**). In those cases, sequential stimulations at different dendritic compartments were performed every 15 s and therefore did not induce a decline in peak amplitude of the evoked signals.

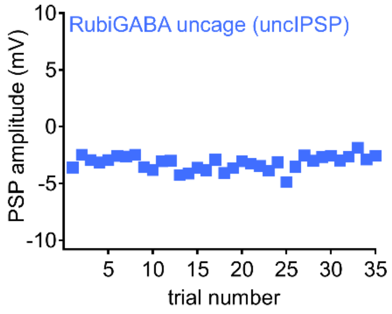

Figure for reviewer #1: UncIPSP amplitude was plotted as function of trial number at 15 s inter-pulse-interval to demonstrate stability in the amplitude of the evoked signals.

3) Fig. 7f, the figure legend does not seem to fit the panel.

We appreciate that the reviewer spotted this mistake. We have corrected it in the legend of the revised **Fig. 7f**.

4) There are several typos, for example in the first paragraph of the introduction.

We apologize for the mistakes. We have carefully read the entire manuscript to correct orthographic mistakes.

Reviewer #2:

We thank reviewer #2 for praising the quality of our study as '*elegantly'* and '*correctly'* performed. However, the reviewer had some questions and comments, which we addressed in the following.

Main Comments:

1) The authors elegantly demonstrate that the EGABA is not uniform in PVIs; having a significantly more depolarized value in proximal, compared to distal locations. These results were obtained correctly with Gramicidin perforated patch experiments. However, in some experiments the authors claimed to use whole-cell recordings and applied an intracellular [Cl-] that resulted in a EGABA of ~63 mV. ('EGABA was kept close to values previously described for dentate gyrus PVIs and GCs, by loading cells during recordings with a pipette solution that resulted in a chloride reversal potential of -63.3 mV.') How did the authors decide what EGABA (proximal or distal) should be 'reproduced' in the whole-cell experiments? What was the intracellular [Cl-] in experiments described in Fig 3-8?

Experiments displayed in **Fig. 1**, were performed with the same intracellular solution for both cell types, which had a calculated reversal potential for chloride of -63.3 mV. This value is located between the E_{GABA} value previously reported for somatic inputs in PVIs (-62 mV; Sauer & Bartos J Neurosci 30, 2010) and GCs (-65.8 mV; Khirug at al., J Neurosci 28, 2008). During the experiments shown in Figure 1, we observed differences in the polarity of the evoked IPSPs in dependence on the RubiGABA uncaging position and in dependence on the cell types. Therefore, we decided to measure E_{GABA} of proximal and distal $GABA_A$ receptor-mediated signals in GCs and PVIs, which are shown in **Fig. 2**. During whole-cell recordings shown in **Fig. 3, 4** and **8,** we used intracellular solutions that would

reflect our, in this study, experimentally measured E_{GABA} values of proximal GABAergic signals for GCs and PVIs. During those recordings, distal dendritic inhibition in PVIs did neither change its polarity, nor its E_{GABA} values (see **Fig. S6b**), indicating strong transporter mechanisms, which keep E_{GABA} constant. See also our antibody labelling against the K-Cl transporter KCC2 in the newly added **Fig. 2g-h** as well as **Fig. S3a**, showing a stronger KCC2 expression at distal than proximal dendrites or the soma of PVIs. When we tried to change distal E_{GABA} in PVIs by loading cells during whole-cell recordings with solution containing different chloride concentrations for more than one hour, we were unable to move distal E_{GABA} to the desired value. In summary, we used pipette solutions to reproduce 'proximal' E_{GABA}. In GCs changes in E_{GABA} were less stable at distal dendrites at recording durations >30 min. We therefore kept the duration of recordings short (<30 min).

2) The authors used realistic cell modelling to predict the dendritic distribution of GGABA. Here, a key parameter, namely the Ra in the different cell types, is unknown. In addition, it is known that the deactivation time constant of the GABAA receptors are very different in these two cell types (much faster in PVIs), suggesting a more pronounced attenuation. Have the authors considered the effects of different inhibitory conductance kinetics?

We thank the reviewer for pointing to these important parameters. Our single cell models were based on currently available data. The axial resistance (R_a) of PVIs was set to 170 Ω cm. This value was obtained from dual somatic and dendritic whole-cell recordings in PVIs (Nörenberg et al., PNAS USA 107, 2000). In GCs it was set to 210 Ω cm in accordance to single cell models performed by Aradi & Holmes, 1999 (J Comput Neurosci 6) and Mateos-Aparicio et al., 2014 (J Physiol Lond 592). This value is close to the experimentally defined value of 194 Ω cm obtained from dual somatic and dendritic whole-cell recordings in GCs (Schmidt-Hieber et al., J Neurosci 27, 2007). However, following the question of the reviewer, we performed new simulations for GCs and compared the effect of a lower R_a value, on the attenuation of dendritically evoked IPSPs (see below **Figure for reviewer #2**). The data show no significant differences in the effect of R_a on signal attenuation in GCs. Due to space limits, we decided not to include the figure in this manuscript but to include the outcome of these simulations in the Methods section on page 13, 3rd paragraph and results section page 6, 3rd paragraph.

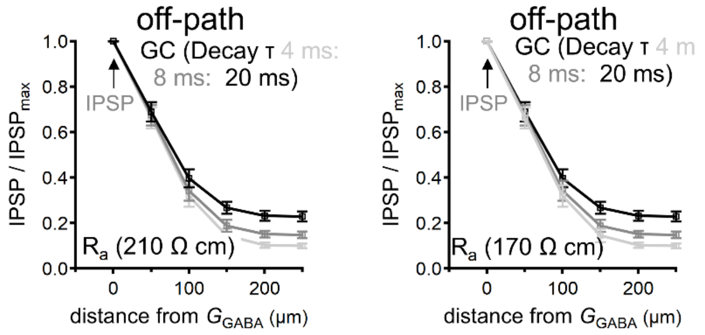

Figure for reviewer #2: The effect of varying the axial resistance (R_a) on the attenuation of dendritically evoked IPSPs with different decay time constants of their underlying conductances in GCs is minimal. Plot shows attenuation of distally evoked IPSPs as a function of distance from the induction site (arrow at distance '0') to the soma (at 250 µm). Different decay time constants of $GABA_A$ receptor mediated conductances were tested (4, 8 and 20 ms). Filled circles with lines represent mean ± SEM.

We agree with the reviewer that $GABA_A$ receptor-mediated signals are ~two-times slower in GCs than PVIs (2.4 and 5.2 ms decay time constant for PVIs and GCs, respectively; Bartos et al., J Neurosci 21, 2001), and could influence the attenuation of signals propagating from their induction site at distal dendrites to the soma. The kinetic

parameters that we used in our models were chosen to better reflect the *in vitro* data obtained in this study. Following the proposal of the reviewer, we altered the kinetic properties of the $GABA_A$ receptor-mediated conductances in our single cell GC and PVI models and measured the attenuation of IPSPs along their propagation to the soma (see new **Fig. S10c** below). We show that reducing the decay time constant to 2 and 4 ms for PVIs and GCs, respectively, increased signal attenuation in both neuron types (due to increased filtering of fast signals). Nevertheless, similar to our original finding, attenuation of distally evoked IPSPs was more pronounced in GCs than in PVIs (0.1 ± 0.011 and 0.17 ± 0.006 $IPSP/IPSP_{max}$ for GCs and PVIs at 250 µm distance from induction site, respectively). We point to these new data in the result section on page 8, 1st paragraph, and add a new plot to **Fig. S10c** of the revised manuscript (see below).

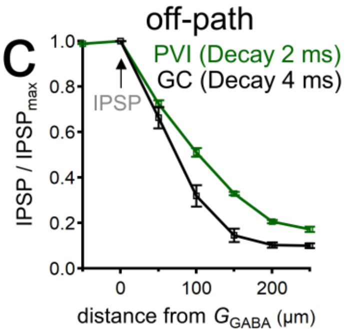

Supplementary Figure 10: (***c***) Attenuation of distally evoked IPSPs was evaluated in GCs and PVIs (3 and 5 cell models, respectively), using rapid decay time constants for $GABA_A$ receptor-mediated conductances (GC: 4 ms, PVI: 2 ms). Squares with lines represent mean ± SEM.

---

3) The plots in figure 6e is hard to read and interpret. The authors should consider changing it to a more conventional 3D plot.

We fully agree with the reviewer's proposal and accordingly changed the 3-D plots in **Fig. 6e** (see below).

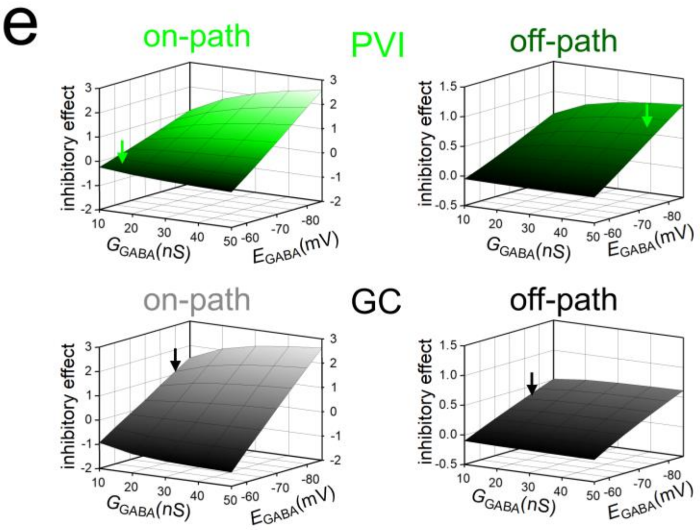

4) The experiments described in figure 7f is very informative, but the number of repetition is very low. The reviewer suggests to increase the 'N' of these experiments preferably in a

way that GCs and PVIs are recorded simultaneously, thus controlling for the effectiveness of the light-evoke GABA release.

We have increased the number of GC and PVI recordings to 9 and 14 cells, respectively, and included them in the revised **Fig. 7f** (see below). Regrettably, simultaneous GC and PVI recordings proved to be difficult, as finding fast-spiking interneurons in slice preparations from injected mice (>P70-day-old) without any intrinsic PVI marker such as PV-GFP resulted to be extremely challenging. Nevertheless, the new data points obtained from single cell recordings show similar results and improved statistical significance of the differences within and between groups. We hope that the reviewer can accept our approach to increase the sample size.

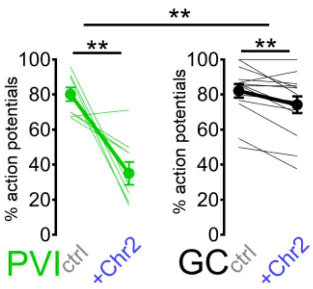

Figure 7f: The perforant path was activated with an extracellular stimulation pipette to evoke single action potentials in GCs and PVIs and the effect of a short overlapping Chr2 pulses on action potential probability is plotted for both neuron types. Circles with lines depict mean ± SEM. Thin lines in panel **f** represent individual experiments. **, *p* ≤ 0.01.

Reviewer #3:

We thank the reviewer for praising our data as '*very interesting*', '*the quality of the data as excellent'* and that the '*conclusions are well supported*' by our results. We are pleased by the reviewer's statement that she/he is '*enthusiastic about this work'*. However, there are some points that the reviewer raised and which we aimed to address in the following.

Main Comments:

1) The biggest criticism is of course, that most data are derived from non-physiological synaptic stimulation. Neither local puffing nor 1p uncaging closely mimics the spatiotemporal spread of synaptically released transmitter and activation of postsynaptic receptors. Indeed, the potential involvement of extrasynaptic receptors in the present study (especially for GABARs) raises the question of whether the results would hold true if only synaptic inhibitory receptors were activated. The optogenetic experiments with SOM-INs mitigate this to some extent. However, this issue is not raised at all in the manuscript. At a minimum, a thorough discussion of this concern should be added. However, it would be much preferable to add more precise stimulation data – e.g., 2p GABA uncaging (though the reviewer acknowledges the technical challenges of this approach).

We thank the reviewer for these important comments. In the case of principal cells, 2P uncaging of glutamate at spines would have increased the likelihood of activating postsynaptic receptors based on the high probability that glutamatergic synapses are

formed at spines. However, the precise location of postsynaptic $GABA_A$ receptors in principal cells is less predictable and it is therefore more difficult to precisely target them. In the case of interneurons, which mostly lack spines, activation of exclusively postsynaptic glutamatergic or GABAergic receptors using 2P uncaging is even more challenging. 2P uncaging requires precise positioning of the laser pulse in the z-plane. In **Figure 1, 3, 4, 5** and **8**, we activated distributed GABAergic inputs over large dendritic distances and different focal planes, making 1P uncaging in our hands the best available tool to induce GABAergic signals in a spatially and temporally controlled manner. To address the concern of the reviewer, we would have needed to perform interneuron-interneuron and interneuron-granule cell paired recordings. However, based on our own experience, slice connectivity particularly from SOMIs and proximally targeting interneurons onto dentate gyrus PVIs is very low (~5%, Savanthrapadian et al., J Neurosci 34, 2014). Thus, such a study is an extreme technical challenge.

We would like to further remark that besides the optogenetic experiment with SOMIs, E_{GABA} measurements were performed by extracellular stimulation of presynaptic interneuron axons targeting the recorded PVIs and GCs and therefore reflect synaptic E_{GABA} values. Moreover, considering that E_{GABA} was the predominant factor that defined inhibitory efficiency, we strongly believe that our results might also hold for pure synaptic GABAergic inputs. Finally, in this revision, we included kinetic properties of GABAergic signals previously defined by paired PVI-PVI and PVI-GC recordings (Bartos et al., PNAS USA 99, 2002) in our single cell models and observed that our original finding of a stronger attenuation of IPSPs in GCs than PVIs remained (see above **Fig. S10c** as response to point 2 of reviewer #2). Nevertheless, we agree with the reviewer that our approach will stimulate both synaptic and extrasynaptic receptors and therefore followed the proposal of the reviewer and discuss this issue on page 10, 1st paragraph. Moreover, we changed the terminology ‘GABAergic inputs’ to ‘GABAergic signals’ throughout the manuscript with the hope that the reviewer can accept our decision.

2) The experiments are done in relatively young animals (P60) to confirm if the basic findings hold true.

One of our basic findings was that distal inhibition in PVIs had a stronger effect in neuronal discharges than in GCs in P19-28-old rats. We observed similar effects of off-path inhibition on the recruitment of PVIs and GCs in adult mice (>P70) using optogenetic activation of SOMI-mediated distal dendritic inhibition. To further confirm that our findings on the E_{GABA} distribution also holds for adult rats, we aimed at performing perforated-patch recordings in slices of >P60-old animals. In case of GCs, we found still a trend for E_{GABA} to be more depolarized in distal dendrites compared to proximal compartments (proximal: 72.5 ± 1.2 mV and distal: -68.7 ± 3.1; 3 GCs). However, in case of PVIs, we were unable to obtain stable perforated-patch recordings. The break-through the membrane happened unexpectedly fast. We therefore followed an alternative approach and performed antibody labelling against the K-Cl co-transporter KCC2 in adult (P71) rats. Interestingly, we observed in both cell types a gradient in the expression KCC2. Normalized intensity of KCC2 antibody labelling was lower at PVI proximal compared to distal dendrites at both young and mature animals (young P21: 1.5 ± 0.2 vs 3.4 ± 0.2 normalized fluorescence intensity, respectively; $p < 0.001$; adult P71: 1.1 ± 0.07 vs 2.0 ± 0.06, $p < 0.001$, two-way ANOVA test with Holm-Sidak pairwise comparison). In contrast, proximal dendritic sections of calbindin-expressing GCs showed a stronger KCC2 expression compared to distal dendrites in both young and adult rats (P21: 2.5 ± 0.1 vs 1.8 ± 0.06; adult P71: 1.9 ± 0.08 vs 1.4 ± 0.5 normalized fluorescence intensity, respectively, $p < 0.001$ for both

comparisons). These the new dat a are included in the revised **Fig. 2g-h** and **Fig. S3a** and in the results section page 4, 3rd paragraph (see also response to point 3c of reviewer #1).

3) The conclusions rest entirely on physiological and computational data. While these results are compelling, they don't distinguish a number of possibilities (differences in receptor subunit composition or synaptic density along the somatodendritic axis). The addition of anatomical data (e.g., immunolabeling gephyrin or better, viral expression of tagged-gepyhrin) to characterize the distribution of putative inhibitory synapses in both cell types would be of enormous benefit.

We fully agree with the reviewer that in order to further support our data it would be beneficial to examine the subunit composition of $GABA_A$ receptor subunits along the somato-dendritic axis of GCs and PVIs of the dentate gyrus, as well as the number and distribution of GABAergic synapses impinging on both neuron types. As a starter, we performed antibody labelling against the α1 and γ2 subunit in slice preparations with PVIs filled during recordings with biocytin, and evaluated their level of colocalization along the somato-dendritic domain. Previous work showed that dentate gyrus GCs express postsynaptic α1, 2 and 3 subunits which confer fast, intermediate and slow deactivation kinetics, and PVIs predominantly α1 and α2 at postsynaptic sites (Farrant & Nusser, Nat Rev Neurosci 6, 2005; Klausberger et al., J Neurosci 22, 2002; Milenkovic et al., Neurosci 254, 2013). They co-assemble with the β2/3 and γ2 subunit. Both tested subunits, α1 and γ2, are members of post- and extrasynaptic $GABA_A$ receptors. Consequently, we observed immunohistochemical labelling at the membrane of the entire somato-dendritic tree. However, comparing the soma-near 50 µm of the proximal dendrite with the 50 µm of the most distal dendritic compartments revealed significant enrichment of the α1 subunit at distal dendrites in PVIs (α1 proximal: 0.4 ± 0.07 vs distal: 0.6 ± 0.09, normalized fluorescence colocalization with biocytin, 5 PVIs; $p < 0.037$, 2-tailed paired t-test). In contrast, γ2 immunolabelling was expressed at similar intensities at proximal and distal dendrites. These data suggest that the composition of $GABA_A$ receptors is different along the somato dendritic axis of PVIs. The data further suggest that dendritically evoked $GABA_A$ receptor-mediated signals may have faster deactivation kinetics than proximally induced ones. This observation fits nicely to our IPSP measurements showing distally evoked IPSPs with faster time courses than proximally evoked ones (rise time, **Fig. 8**). Regrettably, the number of synaptic inputs could not be convincingly determined based on gephyrin antibody labelling and confocal microscopy because of low labelling intensity. Therefore, we could not differentiate between synaptic vs non-synaptic GABAergic receptors. Moreover, we could not use tagged-expression of gephyrin since most of our work has been performed in tissue obtained from rat brains. Nevertheless, previous electronmicroscopical studies showed that the number of GABAergic synapses targeting the somato-dendritic domain of CA1-PVIs is by a factor of 1.54 higher at distal dendrites in *stratum lacunosmum moleculare* than at proximal dendrites in the *pyramidal cell layer* (Gulyas et al., J Neurosci 19, 1999). Although these data have been obtained in CA1-PVIs, they at least show that our hypothesis for dentate gyrus PVIs is in the range of realistic assumptions.

The here obtained data are very exciting, but, in order to gain a proper understanding on how the distribution of the very diverse $GABA_A$ receptor subunits influence inhibition efficiency and dendritic integration would require an independent anatomical study. Such a study should include antibody labelling experiments against the various α and γ subunits, as well as markers for GABAergic synapses in combination with high resolution microscopical techniques for example STED, large volume array tomography and/or electronmicroscopy (e.g. Bloss et al., Neuron 89, 2016). Indeed, we consider using this data

as a basis for an independent study. Therefore, we would like to suggest not continuing on this part for the revision of this manuscript. Following the comments of the reviewer, we now consider in the discussion and the results section the possible mechanisms which may underlie our computational data (number of inputs, different $GABA_A$ receptor subtypes) on page 9, 2nd paragraph and on page 8, 4th paragraph. We hope that the reviewer can accept our strategy and proposal.

4) While some studies are cited, the introduction should really expand a bit on the now substantial literature on dendritic inhibition to set up the overall study. Work from the Higley, Carter, Bonhoeffer, Larkum, and Segev labs have all considered dendritic inhibition extensively.

We thank the reviewer for pointing to these interesting and important papers. Following the suggestion of the reviewer, we have expanded the introduction section on page 2, 3rd paragraph and the discussion to better describe the findings from Marlin & Carter *J Neurosci* **34**, 2014; Müllner et al., *Neuron* **87**, 2015; Murayama et al., *Nature* **457**, 2009; Müller et al., *Neuron* **75**, 2012; Jadi et al., *PLoS Comput Biol* **8,** 2012, and Chiu et al., *Science* **340**, 2013.

REVIEWERS' COMMENTS:

Reviewer #1 (Remarks to the Author):

all my comments were addressed by the authors. I can now recommend publication of this manuscript.

Reviewer #2 (Remarks to the Author):

The reviewer found the revision adequate and has no further suggestion/question.

Reviewer #3 (Remarks to the Author):

Thue authors have done a very nice job responding to my concerns. I am particularly enthusiastic about the new evidence for differential expression and function of KCC2 along the somatodendritic axis.

The reviewers had no further comments or questions to the revised manuscript. We would like to take the opportunity and thank the editor and all three reviewers for their positive evaluation of our work and the constructive comments throughout the revision process.

REVIEWERS' COMMENTS:

Reviewer #1 (Remarks to the Author):

All my comments were addressed by the authors. I can now recommend publication of this manuscript.

Reviewer #2 (Remarks to the Author):

The reviewer found the revision adequate and has no further suggestion/question.

Reviewer #3 (Remarks to the Author):

The authors have done a very nice job responding to my concerns. I am particularly enthusiastic about the new evidence for differential expression and function of KCC2 along the somatodendritic axis.
